# Recircumscription of *Bredia* and resurrection of *Tashiroea* (Sonerileae, Melastomataceae) with description of a new species *T.villosa*

**DOI:** 10.3897/phytokeys.127.36608

**Published:** 2019-07-19

**Authors:** Qiu-Jie Zhou, Jin-Hong Dai, Che-Wei Lin, Tetsuo Denda, Ren-Chao Zhou, Ying Liu

**Affiliations:** 1 State Key Laboratory of Biocontrol and Guangdong Key Laboratory of Plant Resources, School of Life Sciences, Sun Yat-sen University, No. 135, Xin-Gang-Xi Road, Guangzhou 510275, China Sun Yat-sen University Guangzhou China; 2 Herbarium of Taiwan Forestry Research Institute, No. 53, Nan-Hai Road, Taipei 100, Taiwan Herbarium of Taiwan Forestry Research Institute Taipei China; 3 Laboratory of Ecology and Systematics, Faculty of Science, University of the Ryukyus, Senbaru 1, Nishihara, Okinawa 903-0213, Japan University of the Ryukyus Okinawa Japan

**Keywords:** *
Bredia
*, *
Tashiroea
*, Sonerileae, Melastomataceae, morphology, taxonomy

## Abstract

*Bredia* (Melastomataceae) is an Asian genus that extends from central and southern mainland China to Taiwan and the Ryukyu islands. Molecular phylogenetic analyses reveal that the type of *Bredia* is nested in a clade of 20 species, while *Tashiroea*, a genus previously synonymized in *Bredia*, falls in another distantly related clade of 10 species. Our morphological survey shows that the two clades can be distinguished by several diagnostic features including leaf indumentum, texture, leaf surface sculpture under SEM, presence/absence of yellowish uniseriate trichomes, and capsule morphology. Based on molecular and morphological evidence, *Bredia* is recircumscribed and *Tashiroea* is resurrected. Description and a list of species are provided for the two genera with the description of a new species, *T.villosa*.

## Introduction

*Bredia* Blume (Sonerileae, Melastomataceae) was originally described based on *B.hirsuta* Blume (Blume 1849), a species in Taiwan and the Ryukyu islands. Later, Ito and Matsumura (1899) established another genus, *Tashiroea* Matsumura, to accommodate two new species, *T.yaeyamensis* Matsum. and *T.okinawensis* Matsum., also discovered in the Ryukyus. Diels (1924, 1932) recognized both genera. He added one Chinese species to *Tashiroea* and expanded *Bredia* by describing new species and transferring species from *Blastus* Lour., *Otanthera* Blume and *Fordiophyton* Stapf into *Bredia*. Li (1944) followed Diels’s concept of *Bredia* but considered *Tashiroea* to be included within the limits of *Bredia*. He therefore synonymized *Tashiroea*, accommodating its three species within BrediasectionTashiroea (Li 1944). With additional descriptions of new species and transfers of other species (Hooker 1871; Cogniaux 1891; Diels 1924, 1932; Ohwi 1936; Li 1944, 1945; Lauener 1972; Chen 1984a; Yeh et al. 2008; Zhao et al. 2017; Zhou et al. 2018), *Bredia* as currently circumscribed comprises 18–22 species according to different species delimitations (e.g. Chen 1984b; Chen and Renner 2007), and occurs from northern Vietnam and southern mainland China to Taiwan and the Ryukyu islands.

The generic circumscription of *Bredia* has long been problematic. *Bredia* is morphologically closely related to *Phyllagathis* Blume (Blume 1849; Ito and Matsumura 1899; Li 1944; Chen 1984a; Hansen 1992; Chen and Renner 2007), another Asian genus with overlapping geographical range. Delimitation of the two genera was traditionally based on staminal morphology (equal or only slightly unequal, isomorphic vs. dimorphic, subequal, or distinctly unequal stamens), which is rather obscure when species with subequal stamens are concerned. The vague generic boundary is reflected in the taxonomic history of some species that have been moved back and forth between the two genera by different authors (Diels 1932; Merrill and Chun 1940; Li 1944; Chen 1979, 1984b; Hansen 1992; Chen and Renner 2007) and also in two phylogenetic studies based on very limited taxon sampling (Zeng et al. 2016; Zhou et al. 2018).

**Figure 1.**
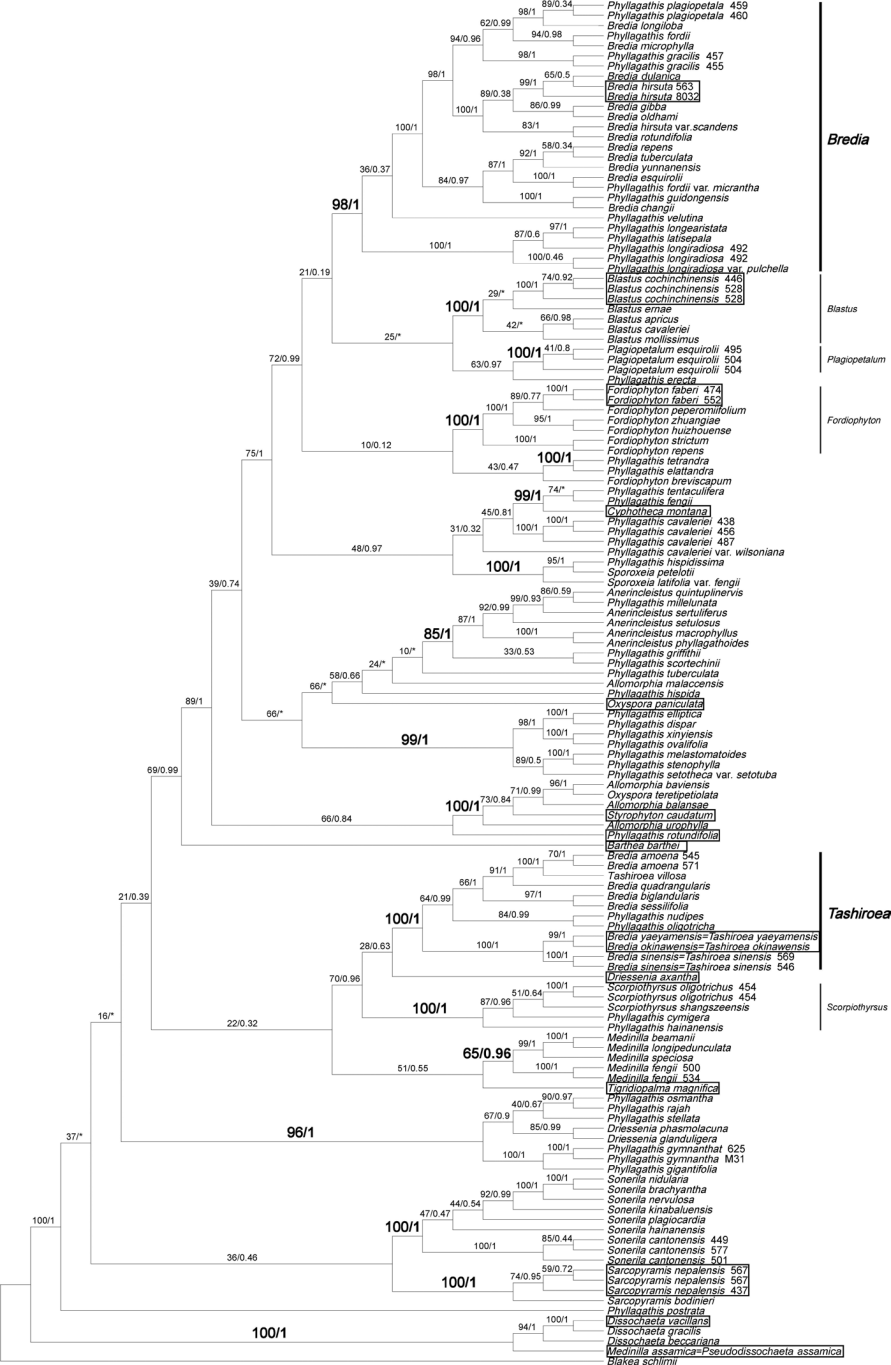
Maximum likelihood phylogenetic tree of Sonerileae/Dissochateae based on combined dataset of nuclear ribosomal internal transcribed spacer and chloroplast *trnV-trnM* sequences, showing the phylogenetic position of *Tashiroea* clade and *Bredia* clade. Numbers on the branches are bootstrap values obtained from maximum likelihood analyses (left) and Bayesian posterior probabilities (right) resulting from Bayesian inference. Boxes denote the types of the genera sampled.

A recent molecular phylogenetic study, with extensive sampling of *Bredia* (19 species) and *Phyllagathis* (35 species), has shed new light on their generic delimitation (Zhou et al. 2019). Staminal characters traditionally used to separate *Bredia* and *Phyllagathis* were shown to be highly homoplasious (Zhou et al. 2019). Both genera as currently defined are not monophyletic. To facilitate discussion, we reconstructed a phylogenetic tree using the combined dataset (nrITS and chloroplast *trnV-trnM*) published in Zhou et al. (2019) plus eight newly sequenced species (see methods). As shown in Fig. 1. *Brediahirsuta*, the type of *Bredia* is clustered within a clade of 20 species, viz. 13 of *Bredia* and seven of *Phyllagathis*, (hereafter referred to as the *Bredia* clade), whereas the two species originally published in *Tashiroea*, *B.yaeyamensis* (Matsum.) H.L. Li (=*T.yaeyamensis*) and *B.okinawensis* (Matsum.) H.L. Li (= *T.okinawensis*), fall in another clade comprising the new species, seven species of *Bredia* and two of *Phyllagathis* (hereafter referred to as the *Tashiroea* clade). The *Bredia* clade is close to *Blastus*, *Fordiophyton* and *Plagiopetalum* Rehder, while the *Tashiroea* clade is most closely related to *Scorpiothyrsus* H.L. Li and *Driesseniaaxantha* Korth. (Fig. 1). These findings are strongly corroborated by our chloroplast phylogenomic analyses (unpublished), indicating that the *Tashiroea* clade is an independent lineage distantly related to the *Bredia* clade. The same conclusion is also reached in a recently published study, although based on limited sampling of species (Kokubugata et al. 2019). Continued use of the non-monophyletic *Bredia* may hinder further study of this group and cause problems in describing new species, which has led us to update its circumscription.

In this paper, we revisited the morphological characters of the *Bredia* and *Tashiroea* clades in search of possible diagnostic characters. The results are presented below. Based on molecular and morphological evidence, *Bredia* is recircumscribed and *Tashiroea* is resurrected. Descriptions and a list of species are provided for the two genera with the description of a new species *T.villosa*. Thirteen new combinations are made.

## Methods

### Molecular experiments and phylogenetic analysis

Eight newly sequenced species were added to the combined dataset (nrITS and chloroplast *trnV-trnM*) published in Zhou et al. (2019), including *Driesseniaglanduligera* Stapf, *D.phasmolacuna* C.W. Lin, *Phyllagathishispidissima* (C. Chen) C. Chen, *P.tentaculifera* C. Hansen, *Scorpiothyrsusshangszeensis* C. Chen, *Brediaokinawensis* (= *Tashiroeaokinawensis*), *B.yunnanensis* (H. Lév.) Diels, and a new species *T.villosa*. All molecular experiments and phylogenetic analysis using bayesian inference and maximum likelihood methods followed Zhou et al. (2019). A complete list of the taxa sampled in this study, their collection localities, voucher information, and GenBank accession numbers are provided in Suppl. material 1: Table S1.

### Morphological comparison

All species of the *Bredia* clade (20 species) and the *Tashiroea* clade (10 species) were examined, including *B.changii* W.Y. Zhao, X.H. Zhan & W.B. Liao, *B.dulanica* C.L. Yeh, S.W. Chung & T.C. Hsu, *B.esquirolii* (H. Lév.) Lauener, *B.gibba* Ohwi, *B.hirsuta*, *B.longiloba* (Hand.-Mazz.) Diels, *B.microphylla* H.L. Li, *B.oldhamii* Hook. f., *B.repens* R.C. Zhou, Q.J. Zhou & Ying Liu, *B.rotundifolia* Yan Liu & C.H. Ou, *B.tuberculata* (Guillaumin) Diels, *B.yunnanensis*, *P.fordii* (Hance) C.Chen, *P.gracilis* (Hand.-Mazz.) C. Chen, *P.guidongensis* K.M. Liu & J. Tian, *P.latisepala* C. Chen, *P.longearistata* C. Chen, *P.longiradiosa* C. Chen, *P.plagiopetala* C. Chen and *P.velutina* (Diels) C. Chen from the former clade, and *B.amoena* Diels, *B.biglandularis* C. Chen, *B.okinawensis* (= *T.okinawensis*), *B.quadrangularis* Cogn., *B.sessilifolia* H.L. Li, *B.sinensis* (Diels) H.L. Li (= *T.sinensis* Diels), *B.yaeyamensis* (= *T.yaeyamensis*), *P.nudipes* C. Chen, *P.oligotricha* Merr. and the new species *T.villosa* from the latter clade. Their habit, indumentum, shape and texture of the leaves, leaf surface sculpture under scanning electronic microscope (SEM), inflorescence type, stamen morphology, capsule morphology, habitat preference and geographical distribution were recorded. Data were obtained via field, herbarium and literature surveys as well as by observing living plants in the facilities of Sun Yat-sen University. Specimens of the two clades (GXMG, GXMI, HNNU, IBG, IBK, IBSC, JJF, KUN, NAS, PE, SYS) or their high-resolution photos (A, BM, CSFI, E, HAST, K, KYO, MO, NTUF, NY, PH, TAI, TAIF, TI, UC, WU) were examined. Habit, shape and texture of the leaves, and inflorescence type were obtained via visual observation. Stamen morphology and capsule morphology were determined by stereomicroscopic (Leica S8APO) examination, leaf epidermal features by desktop SEM (Phenom Pro), and indumentum by both equipment. During observation using Phenom Pro, fresh or dried tissues were directly mounted on stubs and examined without further processing. We failed to obtain the materials of two species recorded in *Bredia*, namely *B.laisherana* C.L. Yeh & C.R. Yeh and *B.violacea* H.L. Li, which were therefore not included in our molecular phylogenetic study. For the two species, we examined their protologue, images of herbarium specimens and color photos from the author of *B.laisherana*. Species circumscriptions basically follow Chen (1984b). Description of the capsule morphology mainly follows Hansen (1992), while the description of one type of trichomes (sessile glands with thin-walled heads) was taken from Wurdack (1986).

### Taxonomy

Diels published several names in Melastomataceae in 1924 and 1932. Although he designated type specimens, he did not specify the place of deposition for them in the protologue. Even if we assume the holotypes existed in the Berlin herbarium (where Diels worked), they were probably destroyed during the Second World War. Therefore, we follow McNeill (2014) and designate lectotypes for those taxa according to the stipulations of Art. 9.12, 9.22 and 9.23 of the Code (Turland et al. 2018). Diels (1932) also lectotypified two names, *Bartheacavaleriei* H. Lév. and *Fordiophytontuberculatum* Guillaumin, without citing the herbaria where they were housed. For *Bartheacavaleriei*, only one specimen of the gathering designated by Diels is known and is in the Royal Botanic Garden Edinburgh herbarium (E). We accept it as the lectotype of this name and cite the page number of Diels’s lectotypification. For *Fordiophytontuberculatum*, multiple specimens of the gathering designated by Diels (1932) are mixtures of more than one taxon. Based on Art. 9.17 (Turland et al. 2018), a lectotype corresponding most closely with the original description is designated for this name. *Tashiroea*, *T.yaeyamensis* and *T.okinawensis* were described simultaneously without designation of a type (Ito and Matsumura 1899). We also lectotypify these names here according to Art. 8.1, 8.2, 10.1, and 10.2 (Turland et al. 2018). In general, we select the herbarium sheet with the best-preserved leaves and flowers for lectotypification. Detailed reasoning where the choice may not be obvious is provided at the end of the taxon being discussed.

## Results

The combined dataset contained 1768 characters. The phylogenetic tree resulted from ML analysis is shown in Fig. 1 with PP and ML bootstrap support values (BS) indicated at nodes. Phylogenetic relationships within Sonerileae/Dissochaeteae are nearly identical to those reported in Zhou et al. (2019). Both *Tashiroea* clade (PP=1.00; BS=100%) and *Bredia* clade (PP=1.00; BS=98%) are well resolved. Among the newly sequenced species, *B.okinawensis* (= *T.okinawensis*) and the new species *T.villosa* are nested in the *Tashiroea* clade, while *B.yunnanensis* is grouped in the *Bredia* clade.

**Figure 2.**
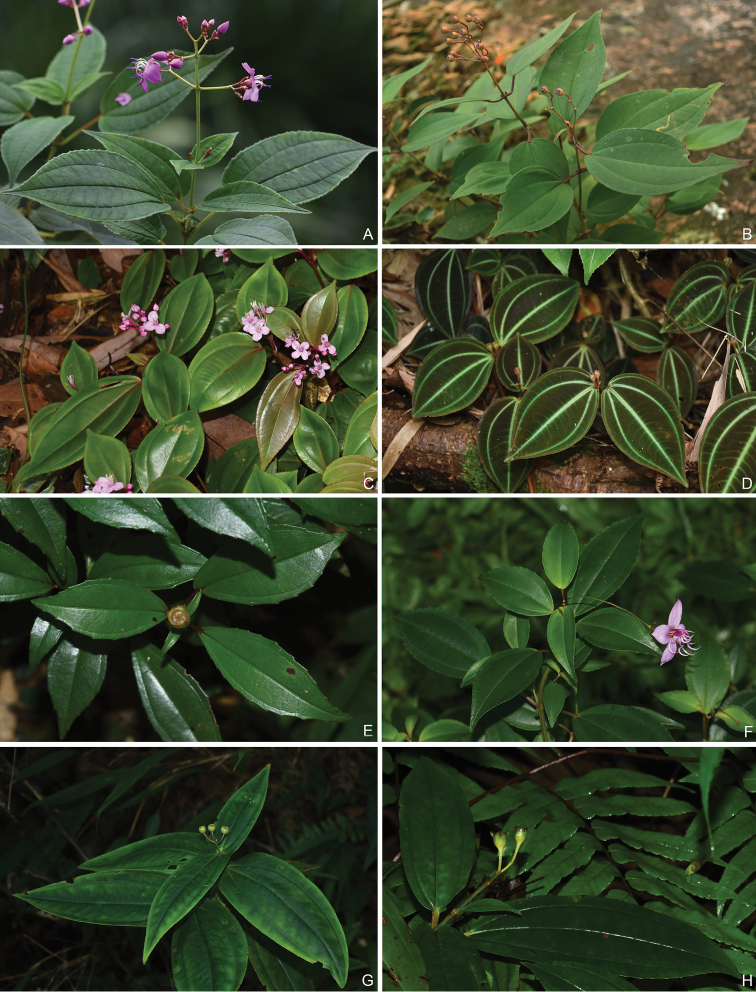
Leaf morphology of the *Tashiroea* clade. **A***T.amoena* (*Y. Liu 571*) **B***T.biglandularis* (*Y. Liu 553*) **C***T.nudipes* (*Y. Liu 435*) **D***T.oligotricha* (*Y. Liu 468*) **E***T.okinawensis* (*Y. Liu 636*) **F***T.quadrangularis* (*Y. Liu 585*) **G***T.sessilifolia* (*Y. Liu 540*) **H***T.yaeyamensis* (*Y. Liu 631*).

**Figure 3.**
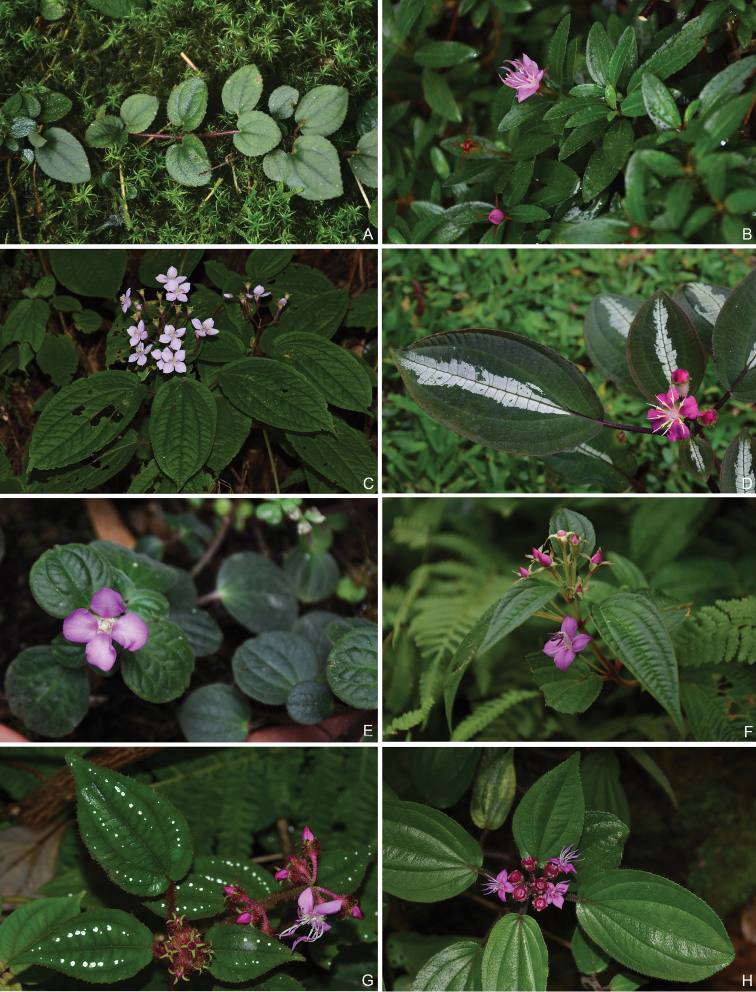
Leaf morphology of the *Bredia* clade. **A***B.changii* (*Y. Liu 548*) **B***B.guidongensis* (*Y. Liu 472*) **C***B.hirsuta* (*Y. Liu 634*) **D**B.longiradiosavar.pulchella (*Y. Liu 485*) **E***B.microphylla* (*Y. Liu 551*) **F***B.plagiopetala* (*Y. Liu 460*) **G***B.tuberculata* (*Y. Liu 629*) **H***B.yunnanensis* (*Y. Liu 627*).

**Figure 4.**
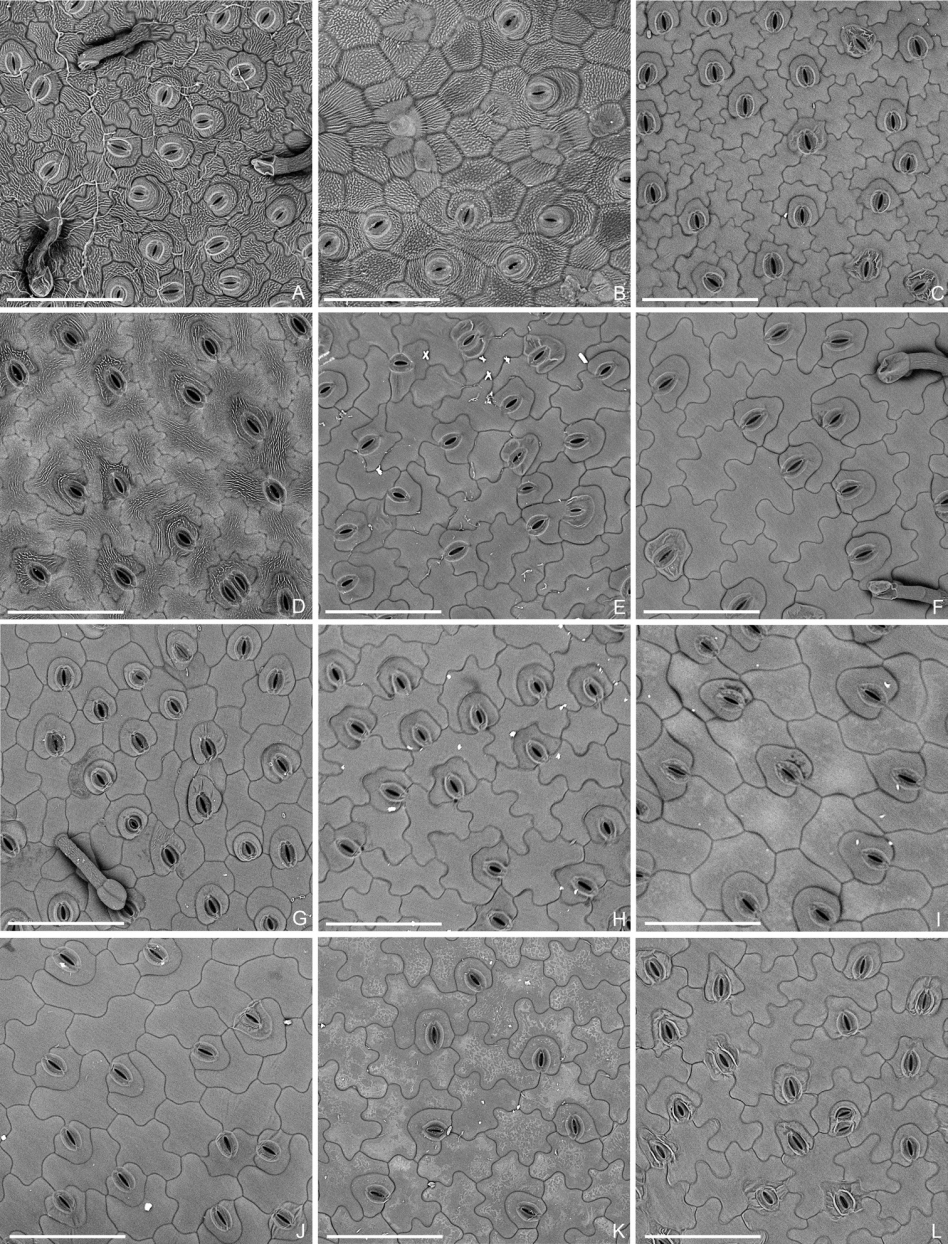
Scanning electron micrographs of lower epidermis. **A–D***Tashiroea* clade, showing presence of furrowed surface sculpture: **A***T.amoena* (*Y. Liu 571*) **B***T.nudipes* (*Y. Liu 435*) **C***T.villosa* (*Y. Liu 568*) **D***T.yaeyamensis* (*Y. Liu 631*) **E–L***Bredia* clade, showing absence of furrowed surface sculpture **E***B.dulanica* (*Y. Liu 565*) **F***B.gibba* (*Y. Liu 566*) **G***B.gracilis* (*Y. Liu 457*) **H***B.hirsuta* (*Y. Liu 563*) **I**B.longiradiosavar.pulchella (*Y. Liu 485*) **J***B.repens* (*Y. Liu 558*) **K***B.tuberculata* (*Y. Liu 629*) **L***B.yunnanensis* (*Y. Liu 627*). Scale bars: 100 μm.

**Figure 5.**
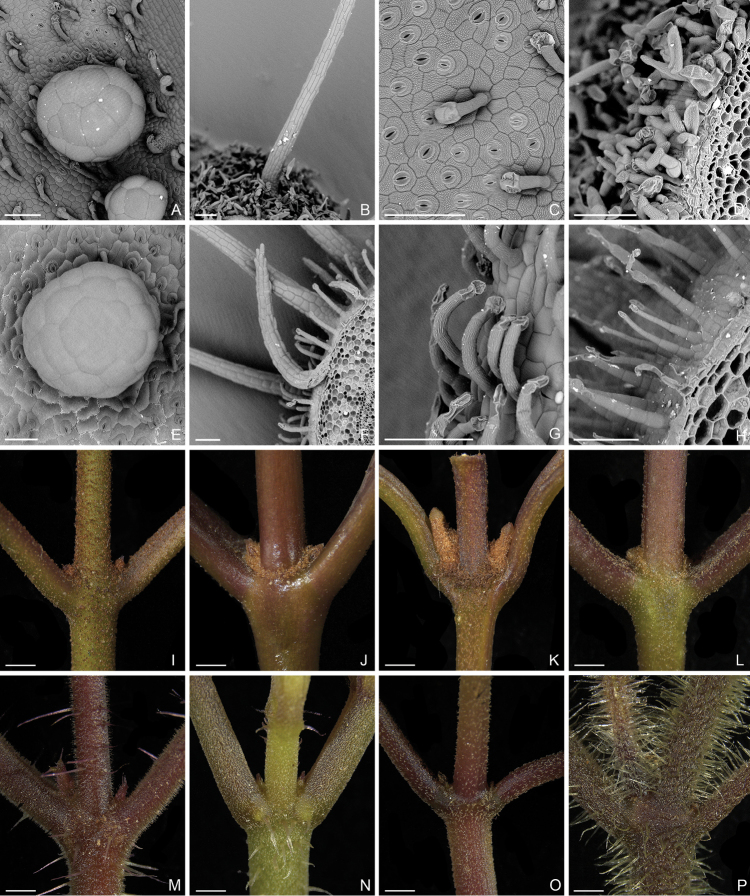
Trichomes in the *Tashiroea* clade and the *Bredia* clade. **A–D** Micrographs of trichomes in the *Tashiroea* clade: **A** sessile gland with thin-walled head in *T.nudipes* (*Y. Liu 435*) **B** multiseriate trichomes in *T.amoena* (*Y. Liu 571*) **C** appressed uniseriate trichomes with glandular heads in *T.nudipes* (*Y. Liu 435*) **D** yellowish uniseriate trichomes (branched or unbranched) in *T.quadrangularis* (*Y. Liu 585*). **E–H** Micrographs of trichomes in the *Bredia* clade: **E** sessile gland with thin-walled head in B.longiradiosavar.pulchella (*Y. Liu 485*) **F** multiseriate trichomes in *B.repens* (*Y. Liu 558*) **G** appressed uniseriate trichomes with glandular heads in *B.gibba* (*Y. Liu 566*) **H** spreading uniseriate trichomes in *B.hirsuta* (*Y. Liu 563*). **I–L** Stereoscopic images of the *Tashiroea* clade, showing buds covered in yellowish uniseriate trichomes **I***T.amoena* (*Y. Liu 571*) **J***T.nudipes* (*Y. Liu 435*) **K***T.quadrangularis* (*Y. Liu 585*) **L***T.yaeyamensis* (*Y. Liu 631*). **M–P** Stereoscopic images of the *Bredia* clade, showing buds without yellowish uniseriate trichomes: **M***B.dulanica* (*Y. Liu 565*) **N***B.gibba* (*Y. Liu 566*) **O***B.gracilis* (*Y. Liu 457*) **P***B.yunnanensis* (*Y. Liu 627*). Scale bars: 100 μm (**A–H**); 1 mm (**I–P**).

**Figure 6.**
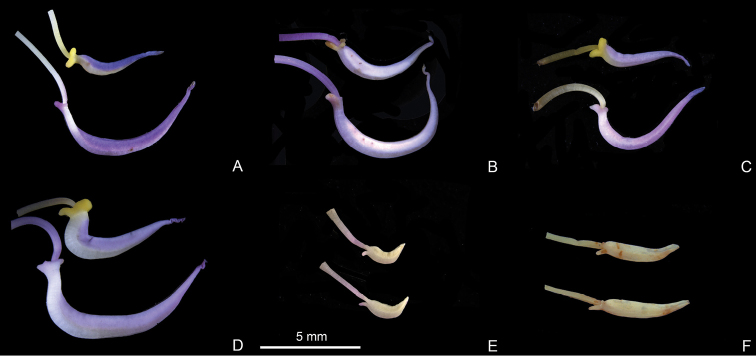
Stamen morphology in the *Tashiroea* clade. **A–D** Dimorphic stamens: **A***T.amoena* (*Y. Liu 571*) **B***T.quadrangularis* (*Y. Liu 585*) **C***T.biglandularis* (*Y. Liu 553*) **D***T.sinensis* (*Y. Liu 569*). **E–F** Isomorphic stamens: **E***T.nudipes* (*Y. Liu 435*) **F***T.oligotricha* (*Y. Liu 468*). Scale bar: 5 mm.

**Figure 7.**
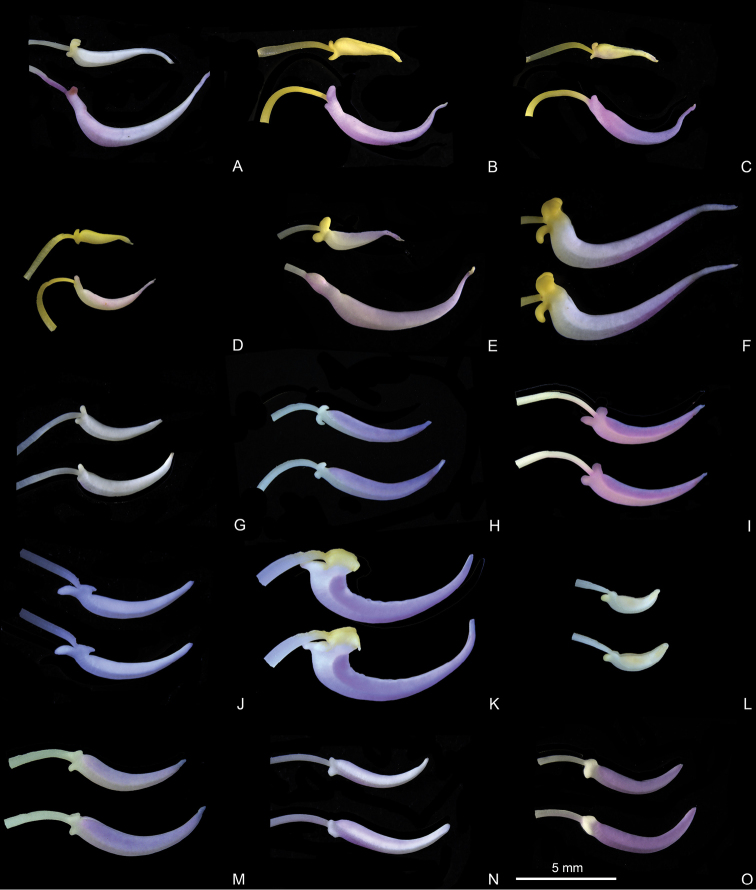
Stamen morphology in the *Bredia* clade. **A–E** Dimorphic stamens: **A***B.esquirolii* (*Y. Liu 587*) **B***B.hirsuta* (*Y. Liu 563*) **C**B.hirsutavar.scandens (*Y. Liu 539*) **D***B.rotundifolia* (*Y. Liu 538*) **E***B.tuberculata* (*Y. Liu 629*). **F–O** Isomorphic stamens: **F***B.fordii* (*Y. Liu 444*) **G**B.fordiivar.micrantha (*Y. Liu 580*) **H***B.gracilis* (*Y. Liu 457*) **I***B.guidongensis* (*Y. Liu 472*) **J***B.longearistata* (*Y. Liu 496*) **K**B.longiradiosavar.pulchella (*Y. Liu 485*) **L***B.microphylla* (*Y. Liu 551*) **M***B.plagiopetala* (*Y. Liu 460*) **N***B.repens* (*Y. Liu 558*) **O***B.yunnanensis* (*Y. Liu 627*). Scale bar: 5 mm.

**Figure 8.**
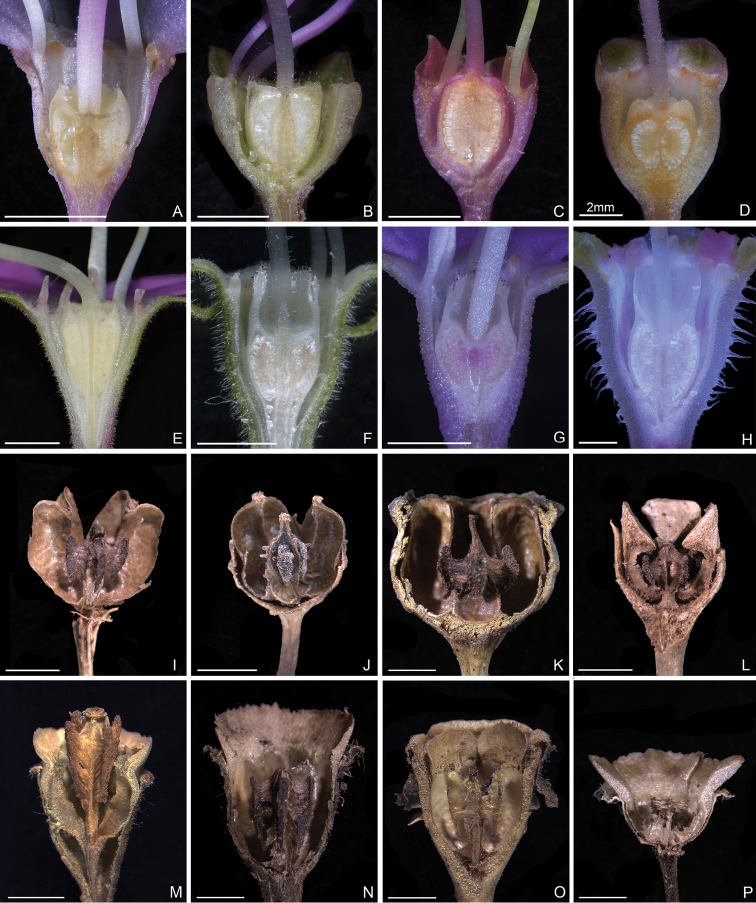
Longitudinal section of ovary (**A–H**) and old fruit (**I–P**). **A–D***Tashiroea* clade, crowned or uncrowned ovaries at anthesis: **A***T.amoena* (*Y. Liu 571*) **B***T.quadrangularis* (*Y. Liu 585*) **C***T.biglandularis* (*Y. Liu 553*) **D***T.sinensis* (*Y. Liu 569*). **E–H***Bredia* clade, crowned ovaries at anthesis: **E***B.hirsuta* (*Y. Liu 563*) **F**B.fordiivar.micrantha (*Y. Liu 580*) **G***B.plagiopetala* (*Y. Liu 459*) **H**B.longiradiosavar.pulchella (*Y. Liu 485*). **I–L***Tashiroea* clade, uncrowned, rarely crowned capsules: **I***T.amoena* (*Y. Liu 571*) **J***T.biglandularis* (*Y. Liu 553*) **K***T.sinensis* (*Y. Liu 569*) **L***T.oligotricha* (*Y. Liu 468*). **M–P***Bredia* clade, crowned capsules: **M***B.dulanica* (*Y. Liu 565*) **N***B.fordii* (*Y. Liu 444*) **O***B.latisepala* (*Y. Liu 557*) **P***B.repens* (*Y. Liu 558*). Scale bars: 2 mm.

Characters of the *Tashiroea* clade (10 species) and the *Bredia* clade (20 species) are summarized in Table 1. Species of the two clades are shrubs or shrublets, except that the latter clade includes some herbs. The mature leaves of species in *Tashiroea* clade are usually glabrous, stiffly papery to leathery and exhibit furrowed surface sculpture under SEM (Figs 2, 4 A–B, D), except that those of the new species *T.villosa* are densely pubescent and villous and without furrowed surface sculpture (Fig. 4C). The mature leaves of species in the *Bredia* clade are usually puberulous and papery and without furrowed surface sculpture (Figs 3, 4 E–L). Species in both clades have sessile glands and uniseriate/multiseriate trichomes with or without glandular heads (Fig. 5A–C, E–H). Yellowish uniseriate trichomes (branched or unbranched) (Fig. 5D), are present only in the *Tashiroea* clade, where they occur on apical and axillary buds and sometimes on young leaves and branches (Fig. 5M–P). Both clades are quite variable in terms of leaf shape (lanceolate to suborbicular), leaf base (cuneate to cordate), inflorescence type (cymose to cymose paniculate), stamen morphology (dimorphic to isomorphic), and staminal appendages (gibbose, tuberculate, or spurred) (Figs 2–3, 6–7). An ovary crown is present in most species of the *Tashiroea* clade at anthesis (Fig. 8A–D), but is usually evanescent, resulting in uncrowned capsules (Fig. 8I–K). Exceptions are two species traditionally placed in *Phyllagathis*. Their crowns are persistent and enlarged, forming crowned capsules with an obpyramidal depression at the top (Fig. 8L). In species of the *Bredia* clade, an ovary crown is present at anthesis (Fig. 8E–H), and persistent and enlarged in old capsule, enclosing an inverted frustum-shaped depression (Fig. 8M–P).

**Table 1.** Comparison of the *Tashiroea* clade and the *Bredia* clade. Potential diagnostic characteristics are indicted in bold.

	*Tashiroea* clade	*Bredia* clade
Habit	Shrubs or shrublets	Shrubs, shrublets or herbs
Indumentum of mature leaf	**Glabrous, rarely pubescent and villous (*T.villosa*)**	**Sparsely to densely puberulous or strigose**
Leaf texture	**Stiffly papery to leathery**	**Papery, rarely submembranous**
Leaf surface sculpture	**Furrowed, rarely not (*T.villosa*)**	**Not furrowed**
Leaf shape	Lanceolate, ovate, elliptic or suborbicular	Lanceolate, ovate, cordate, oblong, elliptic, ovate-orbicular
Leaf base	Cuneate, obtuse, rounded, or subcordate	Cuneate, obtuse, rounded, often cordate
Leaf veins	3–5	5–11, rarely 3 (*B.guidongensis*)
Leaf margin	Entire to serrulate	Entire to serrulate
Indumentum	Sessile gland with thin-walled head; multiseriate trichomes with glandular head or not; appressed uniseriate trichomes with glandular head; **yellowish uniseriate trichomes**	Sessile gland with thin-walled head; multiseriate trichomes with glandular head or not; appressed or spreading uniseriate trichomes with glandular head or not
Inflorescence	Cymose, cymose panicle	Cymose, umbellate, or cymose panicle
Stamens	Dimorphic and isomorphic	Dimorphic and isomorphic
Staminal appendage of dimorphic stamens	Gibbose, tuberculate or spurred at the base of anthers in shorter stamens; connectives decurrent and prolonged, gibbose or tuberculate dorsally in longer stamens	Gibbose, tuberculate or spurred at the base of anthers in shorter stamens; connectives decurrent and prolonged, gibbose, tuberculate, or spurred ventrally in longer stamens
Staminal appendage of isomorphic stamens	Slightly gibbose ventrally at the base of the anthers, spurred dorsally	Gibbose or tuberculate at the base of the anthers, sometimes short spurred dorsally
Ovary top at anthesis	Slightly crowned or uncrowned	Crowned
Capsule	**Ovary crown usually evanescent, capsule uncrowned with rounded or 4-humped top; or ovary crown persistent and enlarged, enclosing an obpyramidal space (in *T.nudipes* and *T.oligotricha*)**	**Ovary crown persistent and enlarged, enclosing an inverted frustum-shaped depression at capsule top**
Habitat	Open or dense forests, slopes, stream banks, alt. 50–2300 m	In forest or along forest margin, stream banks, damp places, alt. 100–2500 m
Distribution	South eastern mainland China, Taiwan, Ryukyu Islands	Central and south mainland China, Taiwan, Ryukyu Islands

Field and literature surveys revealed that species of both *Tashiroea* clade and *Bredia* clade prefer shaded or moist habitats in forests or along forest margin, often along stream banks, from 50 m to 2500 m alt. The *Tashiroea* clade occurs in southeastern mainland China and the Ryukyu islands, while the *Bredia* clade, aside from the above areas, also extends to the central and southwestern Chinese provinces of Guizhou, Hubei, Chongqing, Sichuan and Yunnan.

Of the two species without molecular data, *B.laisherana* is endemic to Taiwan and *B.violacea* occurs in northern Vietnam. The former species has glabrous stems and leaves, and uncrowned ovaries and capsules (Yeh et al. 2008), occurring in dense forests, while the latter has puberulous stems, leaves and inflorescences but no habitat record is available.

## Discussion

### Morphological comparison

Species of the *Tashiroea* clade and *Bredia* clade are similar in habit, habitat preference, most indumentum types, and in having lanceolate, ovate to suborbicular leaves, a cuneate to cordate leaf base, cymose to cymose panicles, dimorphic or isomorphic stamens, and gibbose, tuberculate, or spurred staminal appendages (Figs 2–3, 5–7). Additionally, their range of distribution partly overlaps in southeastern mainland China, and the Ryukyu islands. Therefore, it is quite understandable that Li (1944) considered *Tashiroea* to be congeneric with *Bredia*, where he placed it in synonymy.

However, our survey of morphological characters has shown that the two clades can be distinguished by several diagnostic features, including leaf indumentum, texture, leaf surface sculpture under SEM, presence/absence of yellowish uniseriate trichomes, and capsule morphology. Species of the *Tashiroea* clade differ from those of the *Bredia* clade by the glabrous, stiffly papery to leathery mature leaves with furrowed surface sculpture under SEM (vs. puberulous and papery, without furrowed sculpture) (Figs 2–4), the presence of yellowish uniseriate trichomes on buds (vs. absence) (Fig. 5), and usually uncrowned capsules with a rounded or 4-humped apex, or rarely crowned with an obpyramidal apical depression (vs. capsules crowned, with an inverted frustum-shaped depression) (Fig. 8). *Tashiroeavillosa*, the only exception in the *Tashiroea* clade with densely hairy leaves and without furrowed surface sculpture, nevertheless has yellowish uniseriate trichomes on buds and uncrowned capsules with a 4-humped apex characteristic of this clade. At least some of the above differences, such as indumentum and capsule morphology, had long been noticed by previous authors. Li (1944) established sectionTashiroea in *Bredia*, whereas Hansen (1992) divided *Bredia* into four groups of species, placing *Tashiroea* in a separate group.

The two clades are also morphologically distinguishable from their close relatives. Phylogenetic analyses showed that the *Tashiroea* clade is most closely related to *Scorpiothyrsus* and *Driesseniaaxantha* while *Bredia* clade is closest to *Blastus*, *Fordiophyton*, and *Plagiopetalum* (Fig. 1). Species in the *Tashiroea* clade differ from *Scorpiothyrsus* in the 3–5-veined leaves (vs. 7–9-veined), from *Driesseniaaxantha* in inflorescence terminal (vs. axillary), and from both in inflorescence cymose to cymose panicles (vs. often scorpioid cymose panicles), and ventrally gibbose or tuberculate anthers (vs. 2-setose or 2 band-shaped ventral appendages). The *Bredia* clade is easily distinguished from *Blastus* by having two whorls of stamens (vs. one), from *Fordiophyton* by ventrally appendaged anthers (vs. unappendaged) and the capsule crown usually exserted from the calyx (vs. not exserted), and from *Plagiopetalum* by appendaged anthers and crowned capsules (vs. unappendaged and uncrowned).

### Species without molecular data

Two species previously placed in *Bredia* were not sampled in the molecular phylogenetic studies: *B.laisherana* from Taiwan and *B.violacea* from north Vietnam. *Bredialaisherana* was initially identified as *B.quadrangularis* by Yeh and Yeh (2006) and was later published as a new species (Yeh et al. 2008). It has glabrous stems and leaves, and uncrowned ovaries and capsules (Yeh et al. 2008), which are characteristic of the *Tashiroea* clade. *Brediaviolacea* has puberulous stems, leaves and inflorescences (Li 1945). Li (1945) noted that the general appearance and inflorescence characters of *B.violacea* closely resembled those of *B.hirsuta* (type of *Bredia*). Morphological characters therefore suggest that *B.laisherana* belongs to the *Tashiroea* clade, and *B.violacea* to the *Bredia* clade.

## Conclusion

Morphological evidence and molecular phylogenetic data confirmed that the *Tashiroea* clade and the *Bredia* clade represent two distantly related lineages morphologically well differentiated from each other and from their possible relatives. We therefore resurrect the generic name *Tashiroea* Matsum for the former clade and redefine *Bredia* Blume to include the latter. For the species lack of molecular data, we place *B.laisherana* and *B.violacea* in *Tashiroea* and *Bredia* respectively based on morphology. Species circumscriptions basically follow Chen (1984b). A revision at the species level will be dealt with in another study.

## Taxonomy

### Tashiroea

Taxon classificationPlantaeMyrtalesMelastomataceae

Matsum., J. Coll. Sci. Imp. Univ. Tokyo 12: 489. 1899, emend. R.C. Zhou & Ying Liu

#### Lectotype.

*Tashiroeayaeyamensis* Matsum., J. Coll. Sci. Imp. Univ. Tokyo 12: 489. 1899. (Designated here)

#### Description.

Shrubs or shrublets, erect, rarely creeping in the lower parts. Stems terete or slightly 4-sided, glabrous or glabrescent, rarely densely hairy (in *T.villosa*), terminal and axillary buds pubescent with yellowish uniseriate branched trichomes. Leaves petiolate; leaf blade lanceolate, ovate, elliptic, rarely suborbicular, stiffly papery to leathery, glabrescent when mature, rarely hairy (in *T.villosa*), secondary veins 1 or 2 on each side of midvein, margin remotely serrulate, or almost entire. Inflorescences terminal, few-flowered cymes to cymose panicles; bract minute, rarely to 1–2 cm long (in *T.villosa*), usually caducous. Flowers 4-merous. Hypanthium campanulate, rarely funnel shaped. Calyx lobes repand, crenate or triangular. Petals pink or purplish red, ovate, oblong to suborbicular, more or less oblique, apex acute or acuminate. Stamens 8, unequal or subequal; filaments filiform; anthers dimorphic or isomorphic, subulate to oblong-linear, gibbose, tuberculate or spurred at base, sometime unappendaged adaxially. Ovary half inferior, slightly crowned or uncrowned, ovoid-globular or turbinate, 4-celled. Style filiform; stigma apiculate. Capsule cup-shaped or subglobular, more or less 4-sided, woody, uncrowned, apex rounded or 4-humped, or crown persistent and enlarged, enclosing an obpyramidal space (in *T.nudipes* and *T.oligotricha*). Seeds numerous, minute, cuneate, densely granulate. (Figs 2, 6, 5A–D, I–L, 8A–D, I–L)

#### Distribution.

**Figure 9.**
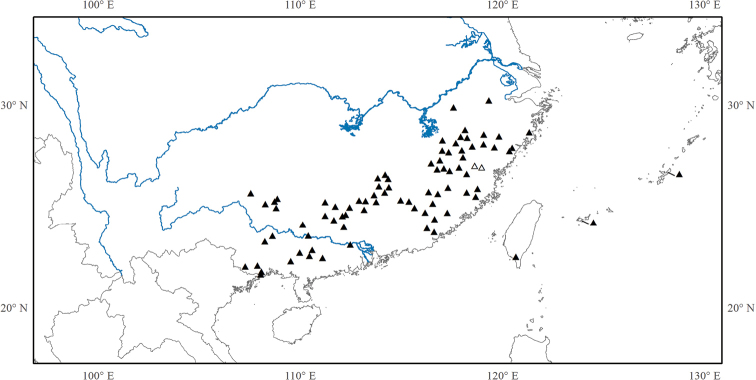
Distribution of *Tashiroea*. Hollow triangles indicate the locations of *Tashiroeavillosa*.

Eleven species, eight in southeastern mainland China (Anhui, Fujian, Guangdong, Guangxi, Guizhou, Hunan, Jiangxi, Zhejiang), one in Taiwan, and two in the Ryukyus (Fig. 9).

Ito and Matsumura (1899) established *Tashiroea* based on *T.yaeyamensis* and *T.okinawensis* without designating a type. The original materials of both species conform to the protologue of *Tashiroea*. We designate *T.yaeyamensis* as the type of *Tashiroea* because this species bears much larger leaves and more flowers than *T.okinawensis*, which may facilitate future molecular and/or morphological analysis of the type materials.

### Species included in *Tashiroea*:

#### Tashiroea amoena

Taxon classificationPlantaeMyrtalesMelastomataceae

(Diels) R.C.Zhou & Ying Liu comb. nov.

urn:lsid:ipni.org:names:77199697-1

Bredia amoena Diels, Notizbl. Bot. Gart. Berlin-Dahlem 9(83): 197–198. 1924 (Basionym). Type: China. Zhejiang: Yentang, 16 Aug 1920, H.H. Hu 30 (lectotype, designated here: A! [A00071979]; isolectotype: UC! [UC231837]).
Bredia chinensis Merr., J. Arnold Arbor. 8(1): 11–12. 1927. Type: China. Zhejiang: Yentang, 16 Aug 1920, H.H. Hu 30 (holotype: UC! [UC231837]; isotype: A! [A00071979]).
Bredia pricei F.P. Metcalf, Lingnan Sci. J. 12: 153–154. 1933. Type: China. W.R. Price 1200A (K! [K001325176]).

#### Tashiroea biglandularis

Taxon classificationPlantaeMyrtalesMelastomataceae

(C. Chen) R.C.Zhou & Ying Liu comb. nov.

urn:lsid:ipni.org:names:77199698-1

Bredia biglandularis C. Chen, Bull. Bot. Res., Harbin 4(3): 39. 1984 (Basionym). Type: China. Guangxi: Dongxing, Lu-bao-shan, 2 Oct 1976, T. Fang 1519 (holotype: GXMI! [GXMI050232]).

#### Tashiroea laisherana

Taxon classificationPlantaeMyrtalesMelastomataceae

(C.L. Yeh & C.R. Yeh) R.C.Zhou & Ying Liu comb. nov.

urn:lsid:ipni.org:names:77199699-1

Bredia laisherana C.L. Yeh & C.R. Yeh, Edinburgh J. Bot. 65(3): 400 (–403; figs 3, 4C–D). 2008 (Basionym). Type: Taiwan, Pingtung, Mt Laisher, on the ridge of a mountain and steep valley and in a dense cloudy forest, 1600–1800 m, 15 Sept 2005, C.L. Yeh & C.R. Yeh 33 (holotype: PPI 065514).

#### Tashiroea nudipes

Taxon classificationPlantaeMyrtalesMelastomataceae

(C. Chen) R.C.Zhou & Ying Liu comb. nov.

urn:lsid:ipni.org:names:77199700-1

Phyllagathis nudipes C. Chen, Bull. Bot. Res., Harbin 4(3): 47. 1984 (Basionym). Type: China. Guangdong: Ruyuan, Wuzhi Shan, 21 May 1973, Guangdong73-101 (holotype: IBSC! [IBSC0003997]).

#### Tashiroea okinawensis

Taxon classificationPlantaeMyrtalesMelastomataceae

Matsum., J. Coll. Sci. Imp. Univ. Tokyo 12: 490. 1899.

Bredia okinawensis (Matsum.) H.L. Li, J. Arnold Arbor. 25: 21. 1944.

##### Type.

Japan. Okinawa: in montosis tractus Kunchan, Apr 1887, Y. Tashiro (5) (lectotype, designated here: TI! [TI00002346]). Additional syntypes: Japan. Okinawa: S. Tanaka 216 (TI! [TI00002347]), Matsumura (TI! [TI00002348]).

#### Tashiroea oligotricha

Taxon classificationPlantaeMyrtalesMelastomataceae

(Merr.) R.C.Zhou & Ying Liu comb. nov.

urn:lsid:ipni.org:names:77199701-1

Phyllagathis oligotricha Merr. Sunyatsenia 1: 74. 1930 (Basionym). Type: China. Guangdong: Lok Chang, 8 Jun 1929, Tso 21016 (holotype: NY! [NY00273007]; isotypes: IBSC! [IBSC0003939, IBSC0003940]).
Phyllagathis anisophylla Diels, Bot. Jahrb. Syst. 65(2–3): 115. 1932. Type: China. Hunan, no precise location, 1926, Hunan Museum 60 (lectotype, designated here: IBSC! [IBSC0003938]; isolectotypes: IBSC! [IBSC0003936, IBSC0003937]).

#### Tashiroea quadrangularis

Taxon classificationPlantaeMyrtalesMelastomataceae

(Cogn.) R.C.Zhou & Ying Liu comb. nov.

urn:lsid:ipni.org:names:77199702-1

Bredia quadrangularis Cogn. Monogr. Phan. 7: 473–474. 1891 (Basionym). Type: South China. Seemann s.n. (LE).

#### Tashiroea sessilifolia

Taxon classificationPlantaeMyrtalesMelastomataceae

(H.L. Li) R.C.Zhou & Ying Liu comb. nov.

urn:lsid:ipni.org:names:77199703-1

Bredia sessilifolia H.L. Li, J. Arnold Arbor. 25: 22. 1944 (Basionym). Type: China. Guangxi: Shang-sze District, Shih Wan Tai Shan, Tang Lung Village, 25 Sept 1934, W. T. Tsang 24346 (holotype: A! [A00071991]; isotype: IBSC! [IBSC0003956]).

#### Tashiroea sinensis

Taxon classificationPlantaeMyrtalesMelastomataceae

Diels, Notizbl. Bot. Gart. Berlin-Dahlem 9: 198. 1924.

Bredia sinensis (Diels) H.L. Li, J. Arnold Arbor. 25: 22. 1944.
Bredia glabra Merr., J. Arnold Arbor. 8: 12. 1927. Type: China. Zhejiang: Pinyong Xian, 11 Jul 1924, Ling Kan 7333 (holotype UC! [UC252284]).

##### Type.

China. Fujian: Chung-an District, 27 Jul 1921, H.H. Hu 1343 (lectotype, designated here: A! [A00073233]).

#### Tashiroea yaeyamensis

Taxon classificationPlantaeMyrtalesMelastomataceae

Matsum., J. Coll. Sci. Imp. Univ. Tokyo 12: 489. 1899.

Bredia yaeyamensis (Matsum.) H.L. Li, J. Arnold Arbor. 25: 21. 1944.
Tashiroea yaeyamensis var. tanakaea Matsum., J. Coll. Sci. Imp. Univ. Tokyo 12: 490. 1899. Type: Okinawa: Yaeyama archipelago, Jul 1890, S. Tanaka 344 (holotype: TI! [TI00002345]).

##### Type.

Japan. Okinawa: Iriomote 1890, S. Tanaka 345 (lectotype, designated here: TI! [TI00002344]). Additional syntype: Japan. Okinawa: in Yaeyama, Aug 1887, Tashiro (TI! [TI00002343]).

#### Tashiroea villosa

Taxon classificationPlantaeMyrtalesMelastomataceae

X.X.Su sp. nov.

urn:lsid:ipni.org:names:77199704-1

**Figure 10.**
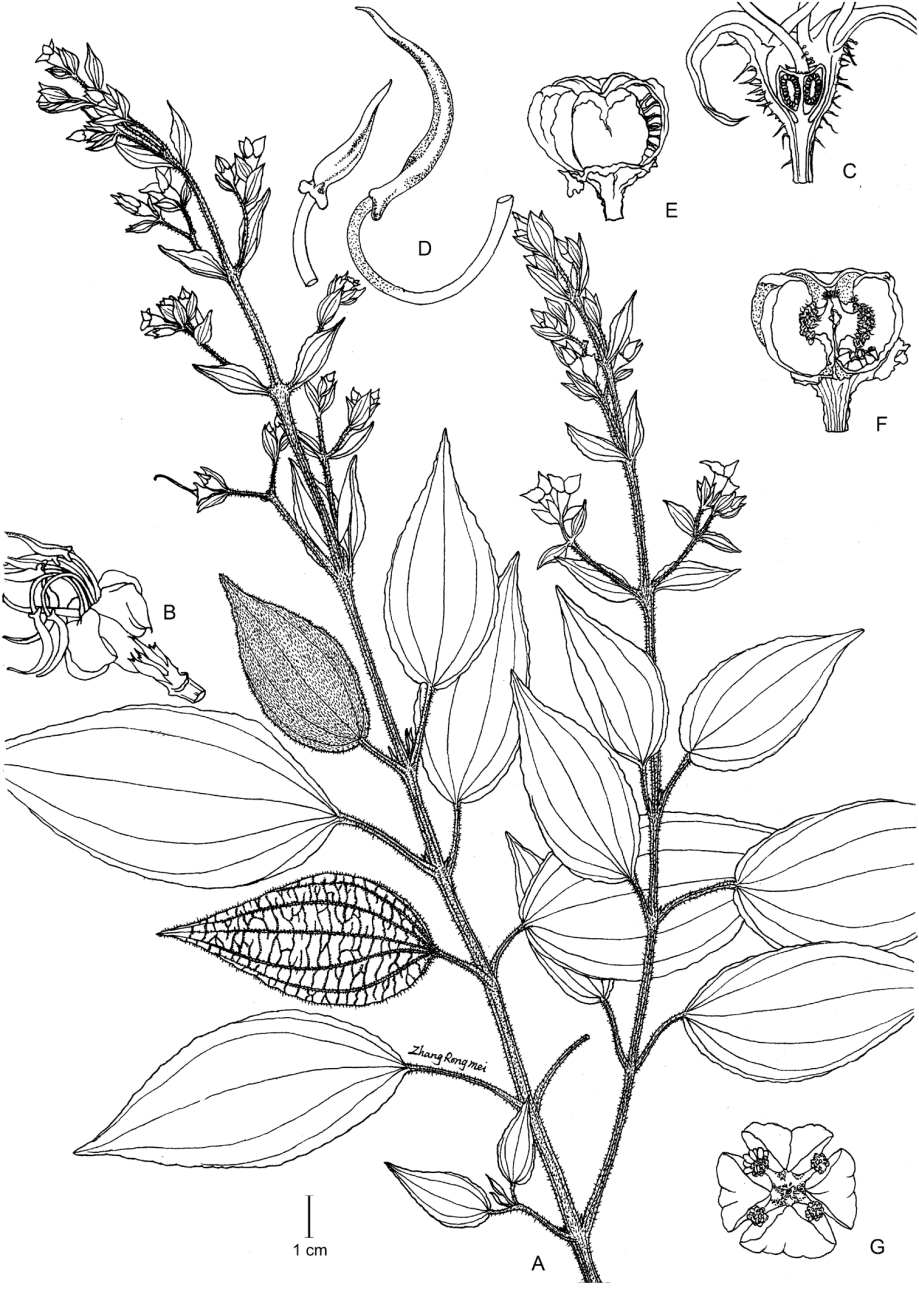
Line illustration of *Tashiroeavillosa* (**A–G**), all from *Y. Liu 568*. **A** Habit **B** Flower (side view) **C** Longitudinal section of flower, showing slightly crowned ovary **D** Dimorphic stamens **E** Capsule (side view) **F** Capsule (longitudinal section) **G** Capsule (top view). Scale bar: 1 cm (**A**).

**Figure 11.**
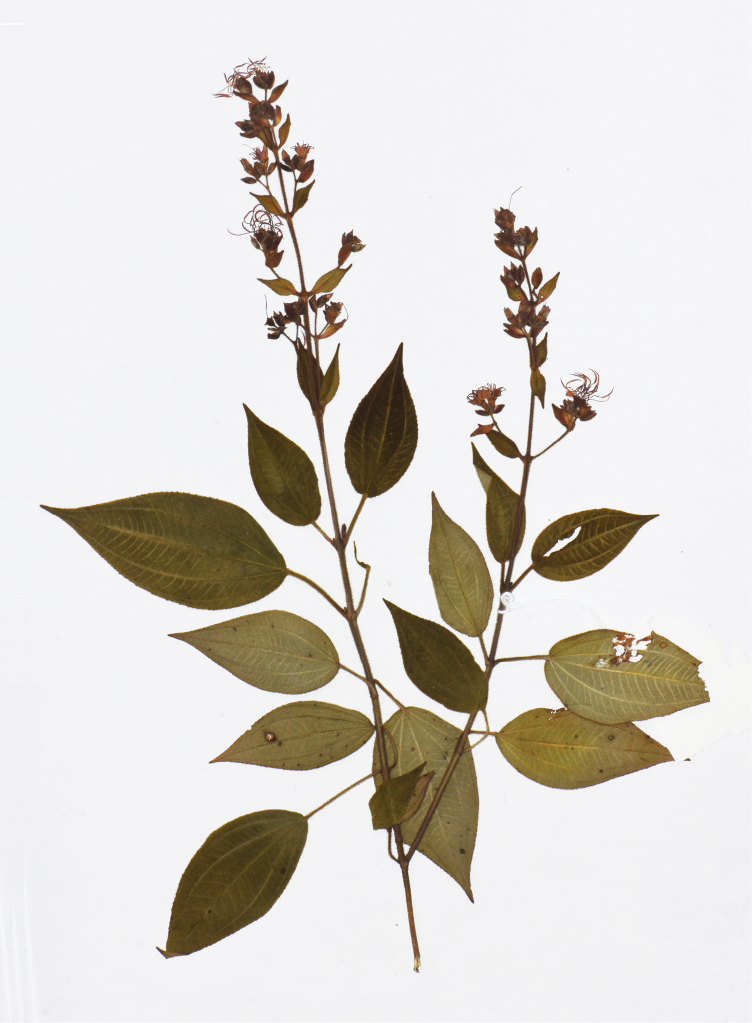
Y. Liu 568 (A) collected from Lingxia, Pingnan County, Fujian, China, holotype of *Tashiroeavillosa*.

**Figure 12.**
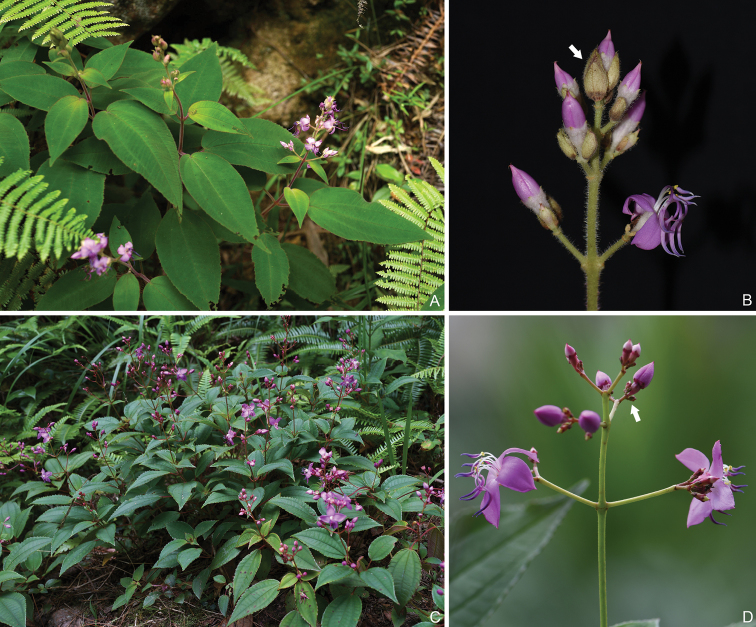
*Tashiroeavillosa* (**A–B**) and *T.amoena* (**C–D**). **A** Habit **B** Inflorescence showing large bracts **C** Habit **D** Inflorescence showing minute bracts. Photographed by Xiang-xiu Su (**A, C**) and Ying Liu (**B, D**).

Figures 10 , 11 , 12A–B

##### Type.

China. Fujian: Pingnan County, Lingxia Town, 1000 m, 16 Jul 2017, Y. Liu 568 (holotype: A!; isotype: SYS!).

##### Diagnosis.

Resembles *T.amoena* in height, leaf size and shape, inflorescence and stamen morphology, while differing from the latter in the dense indumentum covering the whole plant and much larger bracts.

##### Description.

Shrubs or shrublets, 20–60 (–90) cm tall. Stem, leaves, peduncles, bracts, pedicels and hypanthium densely pubescent and villous with multiseriate or sometimes uniseriate glandular or non-glandular trichomes. Stems cylindrical, branchlets slightly 4-sided, sometimes rubescent. Leaves opposite; petiole 1.2–4.5 cm long; leaf blade ovate to ovate-elliptic, 4.2–12 × 1.8–6 cm, papery, abaxial surface pale green, adaxial surface green, secondary veins 3 on each side of midvein, base cordate to rounded, margin ciliate and inconspicuously serrulate, apex acuminate or short acuminate. Inflorescences terminal, cymose, 7–14 × 3.5–6.5 cm, bracts 9–19 × 5–8 mm, deciduous or sometimes persist till anthesis. Pedicels 1–3 mm. Hypanthium short campanulate, 4-sided, 3–5 mm long, calyx lobes broadly triangular, 1 mm long, apex acute. Petals purplish pink or pink, ovate to ovate-oblong, 7–10 × 3.5–5 mm, slightly oblique, apex acute. Stamens 8, dimorphic, unequal. Longer stamens antesepalous, ca. 15 mm long; anthers lanceolate, ca. 8 mm long, geniculate; connective decurrent, slightly prolonged, forming a short spur dorsally. Shorter stamens antepetalous, ca. 8 mm long, anthers lanceolate, ca. 4 mm long, base gibbose ventrally and forming a short spur dorsally. Ovary half inferior, locules 4, apex slightly 4-lobed, margin ciliate with glandular trichomes. Style ca. 0.6 cm long, puberulous with glandular trichomes basally. Capsule cup-shaped; hypanthium ca. 4 × 3.5 mm long; placental column distally entire, placentas non-thready. Seeds numerous, minute, cuneate, granulate. Flowering July-August, fruiting August-October.

##### Etymology.

The specific epithet refers to the dense pubescent and villous indumentum.

##### Distribution and ecology.

*Tashiroeavillosa* is currently known from Pingnan, Jianou and Jianyang, northern Fujian, China (Fig. 9). It often grows in grasses and bushes along streamside at elevations of 900–1400 m.

##### Notes.

*Tashiroeavillosa* is discovered by Mr. Xiang-xiu Su. He is an amateur collector in Fujian who had made an important contribution to the description of this new species. We therefore include him as the author of this name. *Tashiroeavillosa*is the sole species currently known in *Tashiroea* with densely puberulous and villous leaves (vs. glabrous) and smooth leaf surface sculpture (vs. furrowed). It is morphologically and phylogenetically closest to *T.amoena*. The two species are similar in height, leaf size and shape, inflorescence and stamen morphology. *Tashiroeavillosa* is distinct from *T.amoena* in the dense indumentum covering the whole plant (vs. petioles and inflorescences pubescent or sometimes glabrescent) and much larger bracts (9–19 × 5–8 mm vs. 1–2 × 1 mm) in the florescence (Fig. 12). Geographically, *T.amoena* is widely distributed in southeastern China (Anhui, Fujian, Zhejiang, Jiangxi, Guangxi), whereas *T.villosa* occurs in northern Fujian where both species occur. Nevertheless, they have not been found to co-occur within the same habitat.

### Bredia

Taxon classificationPlantaeMyrtalesMelastomataceae

Blume, Mus. Bot. 1: 25. 1849, emend. R.C. Zhou & Ying Liu

#### Type.

*Brediahirsuta* Blume, Mus. Bot. 1(2): 25. f. 4. 1849.

#### Description.

Shrubs, shrublets or herbs, erect, ascending or creeping. Stems terete or more or less 4-sided, sparsely to densely puberulous, rarely glabrescent. Leaves petiolate; leaf blade ovate, cordate, oblong, elliptic, ovate-orbicular, rarely lanceolate, papery, rarely submembranous, sparsely to densely puberulous or strigose, secondary veins 2–5 on each side of midvein, margin serrulate or entire. Inflorescences terminal, umbellate, cymes or cymose panicles. Flowers 4-merous. Hypanthium funnel shaped to campanulate. Calyx lobes conspicuous, linear-lanceolate to triangular. Petals pink or purplish red, ovate to oblong, more or less oblique, apex acute or acuminate. Stamens 8, unequal or subequal; filaments filiform; anthers dimorphic or isomorphic, subulate to oblong-linear, gibbose, tuberculate or spurred at base, rarely unappendaged abaxially. Ovary half inferior, crowned, ovoid, 4-celled. Style filiform; stigma apiculate. Capsule turbinate to cup-shaped, more or less 4-sided, crown persistent and enlarged, enclosing an inverted frustum-shaped depression at capsule apex. Seed numerous, minute, cuneate, densely granulate. (Figs 3, 7, 5E–H, M–P, 8E–H, M–P)

#### Distribution.

**Figure 13.**
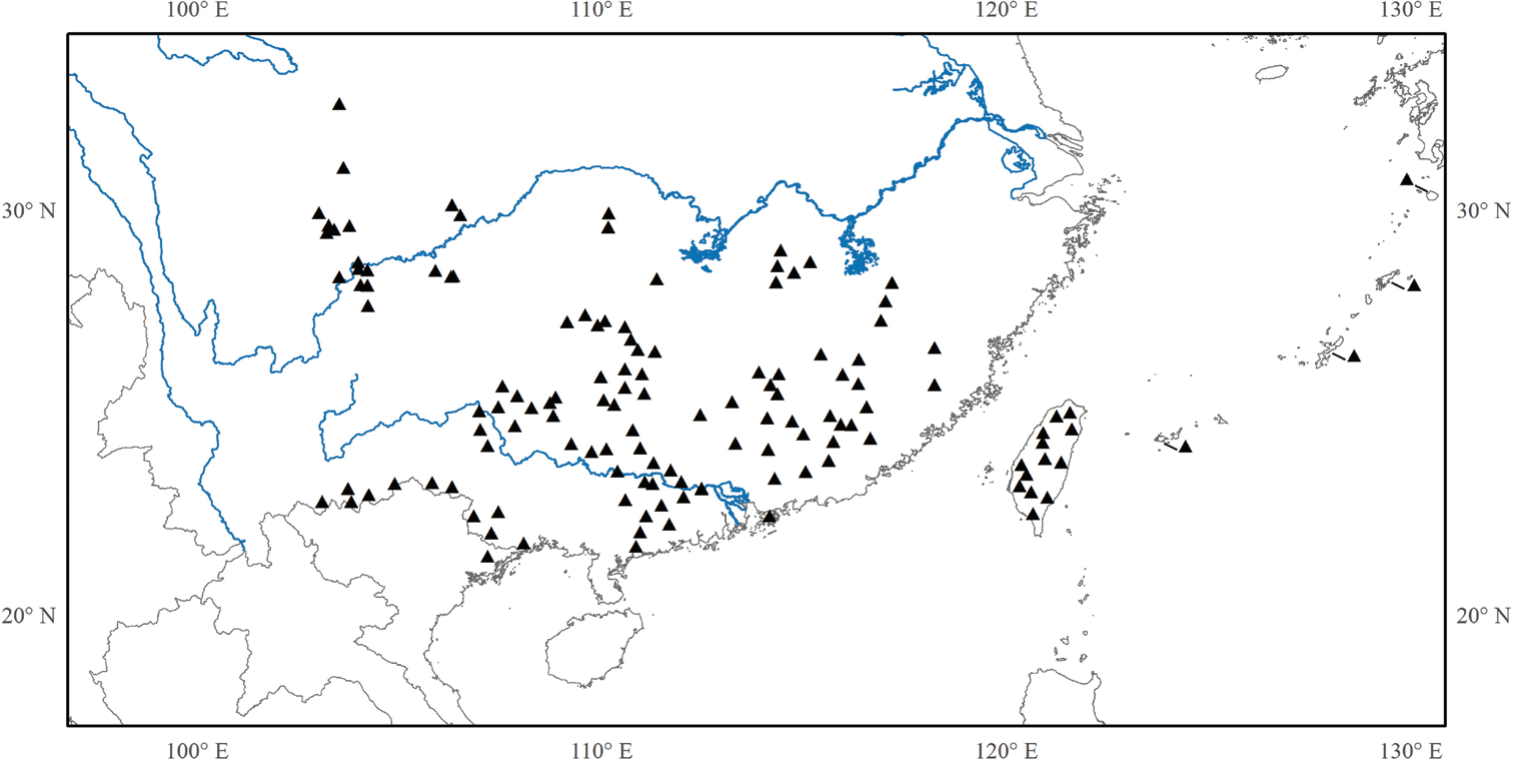
Distribution of *Bredia*.

Twenty-one species: 15 in central and southern mainland China (Fujian, Guangdong, Guangxi, Guizhou, Hubei, Hunan, Jiangxi, Sichuan, Chongqing, Yunnan, Zhejiang), one in north Vietnam, five in Taiwan and one extending to the Ryukyu islands (Fig. 13).

### Species included in *Bredia*:

#### Bredia changii

Taxon classificationPlantaeMyrtalesMelastomataceae

W.Y. Zhao, X.H. Zhan & W.B. Liao, Phytotaxa 307(1): 36–42.

##### Type.

China. Jiangxi: Chongyi County, Niedu Town, 579 m, 1 Aug 2016, W. Y. Zhao et al. LXP-13-22114 (holotype: SYS!; isotype: IBSC!).

#### Bredia dulanica

Taxon classificationPlantaeMyrtalesMelastomataceae

C.L. Yeh, S.W. Chung & T.C. Hsu, Edinburgh J. Bot. 65(3): 395, 399 (figs 2, 4A–B). 2008.

##### Type.

Taiwan, Taitung, Mt Dulan, on the ridge of a mountain, 1000–1200 m, 14 Oct 2007, S.W. Chung, T.C. Hsu & C.R. Yeh 16 (holotype: TAIF! [TAIF348619]; isotypes: TAIF! [TAIF348620, TAIF348621, TAIF348622]).

#### Bredia esquirolii

Taxon classificationPlantaeMyrtalesMelastomataceae

(H. Lév.) Lauener, Notes Roy. Bot. Gard. Edinburgh 31(3): 398–399. 1972.

Barthea esquirolii H. Lév., Repert. Spec. Nov. Regni Veg. 11(301–303): 494. 1913 (Basionym). Type: China. Guizhou: Tchai-choui-ho, July 1909, Esquirol 1581 (holotype: E! [E00090793]).
Bredia cordata H.L. Li, J. Arnold Arbor. 25(1): 24–25. 1944. Type: China. Sichuan: Ya-an, dense forest shade, 686 m, 30 Jul 1939. C. Y. Chiao 1205 (holotype: A! [A00071982]).
Bredia esquirolii var. cordata (H.L. Li) C. Chen, Bull. Bot. Res., Harbin 4(3): 40. 1984.

#### Bredia fordii

Taxon classificationPlantaeMyrtalesMelastomataceae

(Hance) Diels, Bot. Jahrb. Syst. 65(2–3): 110. 1932.

Otanthera fordii Hance, J. Bot. 19: 47. 1881 (Basionym). Type: China. Hong Kong, Jul 1880, C. Ford. herb no. 21099 (lectotype, designated here: BM! [BM000629024]; isolectotype BM! [BM000629025]).
Phyllagathis fordii (Hance) C. Chen, Bull. Bot. Res., Harbin 4(3): 50. 1984.
Bredia sepalosa Diels, 65(2–3): 109–110. 1932. Type: China. Guangxi: Yao shan, 1928, S.S. Sin & K.K. Whang 648 (lectotype, designated here: IBSC! [IBSC0003942]).

#### Bredia fordii var. micrantha

Taxon classificationPlantaeMyrtalesMelastomataceae

(C. Chen) R.C.Zhou & Ying Liu comb. nov.

urn:lsid:ipni.org:names:77199705-1

Phyllagathis fordii var. micrantha C. Chen, Bull. Bot. Res., Harbin 4(3): 50–51. 1984 (Basionym). Type: China. Guizhou: Dushan, in convallibus montanis, 600 m, 22 Aug 1930, Y. Tsiang 6563 (holotype: IBSC! [IBSC0003995]; isotypes: NAS! [NAS00052126] PE! [PE00782806, PE00782810]).

#### Bredia gibba

Taxon classificationPlantaeMyrtalesMelastomataceae

Ohwi, J. Jap. Bot. 12(6): 385–386. 1936.

Bredia penduliflora S.S. Ying, Quart. J. Chin. Forest. 6(1): 167. 1972. Type: Taiwan, Sakanyalan-Tawu, 10 Oct 1972, S.S. Ying 1517 (lectotype, designated here: NTUF! [F00004987]; isolectotypes: NTUF! [F00004986, F00004989, F00004990, F00004991, F00004992, F00004993]).

##### Type.

Taiwan. Pingtung, Sungshan, J. Ohwi s.n. (holotype: KYO! [KYO00022400]).

#### Bredia gracilis

Taxon classificationPlantaeMyrtalesMelastomataceae

(Hand.-Mazz.) Diels, Bot. Jahrb. Syst. 65(2–3): 110. 1932.

Fordiophyton gracile Hand.-Mazz., Akad. Wiss. Wien, Math.-Naturwiss. Kl., Anz. 63: 3, 10. 1926 (Basionym). Type: China. Hunan: Heng Shan, Wukang, 1150–1300 m, 4–8 Aug 1918, Hand.-Mazz. 12380 (holotype: WU! [WU0059491]; isotypes: A! [A00055337] E! [E00090795]).
Phyllagathis gracilis (Hand.-Mazz.) C. Chen, Bull. Bot. Res., Harbin 4(3): 51. 1984.

#### Bredia guidongensis

Taxon classificationPlantaeMyrtalesMelastomataceae

(K.M. Liu & J. Tian) R.C.Zhou & Ying Liu comb. nov.

urn:lsid:ipni.org:names:77199706-1

Phyllagathis guidongensis K.M. Liu & J. Tian, Phytotaxa 263(1): 58–62 (Basionym). Type: China. Hunan: Guidong County, Pule Town, 970 m, 3 Jul 2013, K.M. Liu, R.Y. Yi & L. Peng 24147 (holotype: HNNU; isotypes: HNNU CSFI).

#### Bredia hirsuta

Taxon classificationPlantaeMyrtalesMelastomataceae

Blume, Mus. Bot. 1(2): 25. f. 4. 1849.

##### Type.

Japan. K. Ito s.n. (lectotype, designated here: L! [L0170980]).

In the protologue, Blume (1849) cited no specimen but the name “*Rhexiafasikan*” (should be *Rhexiahasikan*, see below) attached to a specimen from Von Siebold’s herbarium. Ohba and Akiyama (pers. comm.) discovered one sheet with the name “*Rhexiahasikan*” in the herbarium in Leiden (L0170980). The sheet contains two collections, a smaller one covered by paper on the upper part and a larger one with four branches on the middle and lower part. According to Ohba (pers. comm.), the epithet “hasikan” comes from the Japanese name of *B.hirsuta*, viz. “Hashikan-boku”. This sheet is regarded as the type material of *B.hirsuta*. We here designate the larger collection on the sheet as the lectotype of *B.hirsuta*.

#### Bredia hirsuta var. scandens

Taxon classificationPlantaeMyrtalesMelastomataceae

Ito & Matsum., J. Coll. Sci. Imp. Univ. Tokyo 12: 487. 1898.

Bredia scandens (Ito & Matsum.) Hayata. J. Coll. Sci. Imp. Univ. Tokyo 30(1): 114. 1911.

##### Type.

Taiwan, inter Suiteiryō et Niki, C. Owatari, Jan 1898 (the date “1896” cited in the protologue is probably erroneous) (lectotype, designated here: TI! [TI00002337]; isolectotype: TI!, [TI00002339]).

#### Bredia latisepala

Taxon classificationPlantaeMyrtalesMelastomataceae

(C. Chen) R.C.Zhou & Ying Liu comb. nov.

urn:lsid:ipni.org:names:77199707-1

Phyllagathis latisepala C. Chen, Bull. Bot. Res., Harbin 4(3): 53–54. 1984 (Basionym). Type: China. Hubei: Hefeng, ad pedes montis calcareo, 18 Sept 1958, H.J. Li 6451 (holotype: IBSC! [IBSC0003996]; isotype: PE! [PE00025692]).

#### Bredia longearistata

Taxon classificationPlantaeMyrtalesMelastomataceae

(C. Chen) R.C.Zhou & Ying Liu comb. nov.

urn:lsid:ipni.org:names:77199708-1

Phyllagathis longearistata C. Chen, Bull. Bot. Res., Harbin 4(3): 52–53. 1984 (Basionym). Type: China. Guangxi: Hechi, prope rivulos in convallibus montanis, 19 May 1928, L.H. Chun 91861 (holotype: IBK! [IBK00190677]; isotype: IBK! [IBK00190678]).

#### Bredia longiloba

Taxon classificationPlantaeMyrtalesMelastomataceae

(Hand.-Mazz.) Diels, Bot. Jahrb. Syst. 65(2–3): 111. 1933.

Fordiophyton gracile var. longilobum Hand.-Mazz., Akad. Wiss. Wien, Math.-Naturwiss. Kl., Anz. 63: 3, 10. 1926 (Basionym). Type: China. Jiangxi: between Ningdu and Ki-an, T.H. Wang 493 (holotype: WU! [WU0059490]).

#### Bredia longiradiosa

Taxon classificationPlantaeMyrtalesMelastomataceae

C. Chen ex Govaerts, World Checkl. Seed Pl. 2(1): 13. 1996.

Phyllagathis longiradiosa C. Chen, Bull. Bot. Res., Harbin 4(3): 51. 1984 (Basionym). Type: Based on Bartheacavaleriei H. Lév.
Barthea cavaleriei H. Lév., Repert. Spec. Nov. Regni Veg. 8(160–162): 61. 1910. Type: China. Guizhou: near Mou-you-sé, J. Cavalerie 1552 (lectotype, designated by Diels 1932, pg. 110: E! [E00090789]).
Fordiophyton cavaleriei (H. Lév.) Guillaumin, Bull. Soc. Bot. France 60: 275. 1913.
Bredia cavaleriei (H. Lév.) Diels, Bot. Jahrb. Syst. 65(2–3): 110. 1932.
Bredia longiradiosa C. Chen, Fl. Yunnan 2: 105, f. 27, 1–5. 1979. nom. inval. (reference to place of publication of basionym not provided).

#### Bredia longiradiosa var. pulchella

Taxon classificationPlantaeMyrtalesMelastomataceae

(C. Chen) R.C.Zhou & Ying Liu comb. nov.

urn:lsid:ipni.org:names:77199709-1

Phyllagathis longiradiosa var. pulchella C. Chen, Bull. Bot. Res., Harbin 4(3): 52. 1984 (Basionym). Type: China. Guangxi: Daxin, Longjin, 4 May 1959, F.F. Huang 3596 (holotype: GXMI! [GXMI050237]).

##### Notes.

Three gatherings were cited in the protologue of *Bartheacavaleriei*, viz. Cavalerie 1552, Esquirol 1581 and Esquirol 215, without designation of a type. Two of the syntypes, Esquirol 1581 and 215 were later cited as the types of *Bartheaesquirolii* H. Lév. (1913) and *Bartheablinii* H. Lév. (1913), respectively. Guillaumin (1913) transferred *Bartheacavaleriei* to *Fordiophyton* citing only Cavalerie 1552 under the new combination *F.cavaleriei* (H. Lév.) Guillaumin. Diels (1932) formally designated Cavalerie 1552 as the lectotype and published a new combination *Brediacavaleriei* (H. Lév.) Diels. However, this name is an illegitimate later homonym of *B.cavaleriei* H. Lév. & Vaniot (see Art. 53.1). Chen (1979) published the replacement name *B.longiradiosa* C. Chen for this species, but he did not provide a full reference of the basionym, which makes the name invalid (see Art. 41.1). Chen (1984b) subsequently transferred the species to *Phyllagathis*. As the epithet *cavaleriei* is also not available in *Phyllagathis*, a new name *P.longiradiosa* C. Chen was published together with the description of a new variety, P.longiradiosavar.pulchella C. Chen. There are three effectively published names for this species in *Bredia*. With *B.cavaleriei* (H. Lév.) Diels illegitimate and *B.longiradiosa* C. Chen invalid, the only legitimate name, *B.longiradiosa* C. Chen ex Govaerts is adopted as the correct name for this species.

#### Bredia microphylla

Taxon classificationPlantaeMyrtalesMelastomataceae

H.L. Li, J. Arnold Arbor. 25(1): 23. 1944.

##### Type.

China. Guangxi: Guilin District, Chi-fen Shan, Xichang Cun and vicinity, W.T. Tsang 28432 (holotype: A! [A00071988]; isotype: IBSC! [IBSC0003950]).

#### Bredia oldhamii

Taxon classificationPlantaeMyrtalesMelastomataceae

Hook. f., Icon. Pl. 11: 68, pl. 1085. 1871.

Bredia oldhamii var. ovata Ohwi, J. Jap. Bot. 12(9): 661–662. 1936. Type: Taiwan, Taitung, in open forest, forest margin, 100–1200 m, J. Ohwi 425 (holotype: KYO).

##### Type.

Taiwan, near Tamsuy, Jan 1864, R. Oldham 118 (holotype: K! [K000978944]; isotype: GH! [GH00071989] P! [P02274733] US! [US00120439]).

#### Bredia plagiopetala

Taxon classificationPlantaeMyrtalesMelastomataceae

(C. Chen) R.C.Zhou & Ying Liu comb. nov.

urn:lsid:ipni.org:names:77199710-1

Phyllagathis plagiopetala C. Chen, Bull. Bot. Res., Harbin 4(3): 44–45. 1984 (Basionym). Type: China. Hunan: Xinning, Ziyun Shan, in dense silvis apice montium, 800 m, 11 Jul 1959, P.C. Tam 63423 (holotype: IBK! [IBK00127587]).

#### Bredia repens

Taxon classificationPlantaeMyrtalesMelastomataceae

R.C. Zhou, Q.J. Zhou & Ying Liu, Syst. Bot. 43(2): 549. 2018.

##### Type.

China. Hunan: Sangzhi County, from Shayuan to Nanmuping village, 430–470 m, 11 Nov 2016, Y. Liu 558 (holotype: SYS!; isotypes: A! SYS!).

#### Bredia rotundifolia

Taxon classificationPlantaeMyrtalesMelastomataceae

Yan Liu & C.H. Ou, Quart. J. Chin. Forest. 9(2): 118, f. 1. 1976.

##### Type.

Taiwan, Chiayi, Juili, Ou 2869 (holotype: NCUF).

#### Bredia tuberculata

Taxon classificationPlantaeMyrtalesMelastomataceae

(Guillaumin) Diels, Bot. Jahrb. Syst. 65(2–3): 111. 1932.

Fordiophyton tuberculatum Guillaumin, Notul. Syst. (Paris) 2(11): 326. 1913 (Basionym). Type: China. Yunnan: Tchen fong chan, Delavay 5053 (lectotype, designated here: E! [E00285959]; isolectotypes: P! [P02274731], P! [P02274729] individual on the right side of the sheet).
Bredia omeiensis H.L. Li, J. Arnold Arbor. 25(1): 24. 1944.Type: China. Sichuan: Emei Shan, 1100 m, 21 Aug 1937, Y.S. Liu 1080 (holotype: A! [A00071990]; isotype: LBG! [LBG00089612]).

##### Notes.

Three gatherings were cited in the protologue of *Fordiophytontuberculatum*, viz. Delavay 5053, Ducloux 2192 and Wilson 4906. Diels (1932) designated Delavay 5053 as the type without citing a specific herbarium. We located four duplicates of Delavay 5053 (E00285959, P02274729, P02274730, P02274731). Close examination of the specimens revealed that this gathering is a mixture of *B.tuberculata* and *B.yunnanensis*. Individuals on E00285959, P02274731 and the one on the right side of P02274729 conform to the protologue of *B.tuberculata* in the adaxially white punctate leaves and unequal and dimorphic stamens. The remaining four individuals (three on P02274730 and one on the left side of P02274729) conform to the holotype of *B.yunnanensis* in the absence of white spots on their leaves and the subequal and isomorphic stamens. According to Art. 9.11, 9.12, 9.14, and 9.17 (Turland et al. 2018), the lectotypification by Diels should be accepted and further narrowed to a single specimen that corresponds most closely with the original description. We therefore designate E00285959 as the lectotype as it conforms to the description and contains many well-preserved leaves and flowers.

#### Bredia violacea

Taxon classificationPlantaeMyrtalesMelastomataceae

H.L. Li, J. Arnold Arbor. 26(1): 120. 1945.

##### Type.

Vietnam. Tonkin: Tian-yen, Ho Yung Shan & vicinity, 13 Oct–22 Nov 1940, W.T. Tsang 30751 (holotype: A! [A00071992]).

#### Bredia velutina

Taxon classificationPlantaeMyrtalesMelastomataceae

Diels, Bot. Jahrb. Syst. 65(2–3): 109. 1932.

Phyllagathis velutina (Diels) C. Chen, Bull. Bot. Res., Harbin 4(3): 51. 1984.

##### Type.

China. Yunnan: Mengzi, 1000–2300 m, A. Henry 13479 (lectotype, designated here: K! [K000867582]; isolectotypes: A! [A00055334] NY! [NY00221472]).

#### Bredia yunnanensis

Taxon classificationPlantaeMyrtalesMelastomataceae

(H. Lév.) Diels, Bot. Jahrb. Syst. 65(2–3): 111. 1932.

Blastus yunnanensis H. Lév., Repert. Spec. Nov. Regni Veg. 11(286–290): 300–301. 1912 (Basionym). Type: China. Yunnan: Vallée de Long-Ky, pied des rochers humides, 700 m, Aug 1911, Maire s.n. (holotype: E! [E00285956]).
Blastus mairei H. Lév., Repert. Spec. Nov. Regni Veg. 11(286–290): 300. 1912. Type: China. Yunnan: Bord des eaux, Vallée de Long-Ky, 700 m, Jul 1911, Maire s.n. (holotype: E! [00285957]).

## Supplementary Material

XML Treatment for Tashiroea

XML Treatment for Tashiroea amoena

XML Treatment for Tashiroea biglandularis

XML Treatment for Tashiroea laisherana

XML Treatment for Tashiroea nudipes

XML Treatment for Tashiroea okinawensis

XML Treatment for Tashiroea oligotricha

XML Treatment for Tashiroea quadrangularis

XML Treatment for Tashiroea sessilifolia

XML Treatment for Tashiroea sinensis

XML Treatment for Tashiroea yaeyamensis

XML Treatment for Tashiroea villosa

XML Treatment for Bredia

XML Treatment for Bredia changii

XML Treatment for Bredia dulanica

XML Treatment for Bredia esquirolii

XML Treatment for Bredia fordii

XML Treatment for Bredia fordii var. micrantha

XML Treatment for Bredia gibba

XML Treatment for Bredia gracilis

XML Treatment for Bredia guidongensis

XML Treatment for Bredia hirsuta

XML Treatment for Bredia hirsuta var. scandens

XML Treatment for Bredia latisepala

XML Treatment for Bredia longearistata

XML Treatment for Bredia longiloba

XML Treatment for Bredia longiradiosa

XML Treatment for Bredia longiradiosa var. pulchella

XML Treatment for Bredia microphylla

XML Treatment for Bredia oldhamii

XML Treatment for Bredia plagiopetala

XML Treatment for Bredia repens

XML Treatment for Bredia rotundifolia

XML Treatment for Bredia tuberculata

XML Treatment for Bredia violacea

XML Treatment for Bredia velutina

XML Treatment for Bredia yunnanensis

## Supplementary materials

Supplementary material 1 Table S1. Source of materials studied and GenBank accession numbers for nrITS and chloroplast intergenic spacer *trnV-trnM* Data type: molecular data https://binary.pensoft.net/file/318136This dataset is made available under the Open Database License (http://opendatacommons.org/licenses/odbl/1.0/). The Open Database License (ODbL) is a license agreement intended to allow users to freely share, modify, and use this Dataset while maintaining this same freedom for others, provided that the original source and author(s) are credited.Qiu-Jie Zhou, Jin-Hong Dai, Che-Wei Lin, Tetsuo Denda, Ren-Chao Zhou, Ying Liu
