# Supplementary material for: Recircumscription of Bredia and resurrection of Tashiroea (Sonerileae, Melastomataceae) with description of a new species T.villosa
Source: PhytoKeys. 2019 Jul 19;127:121–50. doi: 10.3897/phytokeys.127.36608 (PMC6661266; doi:10.3897/phytokeys.127.36608)
Supplement: Supplementary material 1 [file phytokeys-127-121-s001.docx]

**Table. S1.** Source of materials studied and GenBank accession numbers for nrITS and chloroplast intergenic spacer *trnV-trnM*. Sequences newly obtained for this study are indicated in asterisk.

| Taxon | Locality | Voucher | ITS | *trnV-trnM* |
| --- | --- | --- | --- | --- |
| Tr. Blakeeae |  |  |  |  |
| *Blakea* *schlimii* (Naudin) Triana | - | - | AY460441 | NC_031877 |
| Tr. Dissochaeteae |  |  |  |  |
| *Dissochaeta beccariana* Cogn. | Kuching, Malaysia | *Zhou et al. M34* (SYS) | MG644477 | MG644588 |
| *D. gracilis* Blume | Java, Indonesia | *Liu 601* (SYS) | MG644479 | MG644590 |
| *D. vacillans* Blume | Java, Indonesia | *Liu 602* (SYS) | MG644478 | MG644589 |
| *Medinilla assamica* (C.B. Clarke) C. Chen (=*Pseudodissochaeta assamica (C.B.Clarke) Nayar*) | Hekou, Yunnan, China | *Liu 516* (SYS) | MG644480 | MG644591 |
| *M. beamanii* Regalado | Sabah, Malaysia | *Zhou et al. M1* (SYS) | MG644481 | MG644592 |
| *M. fengii* (S.Y. Hu) C.Y. Wu & C. Chen | Malipo, Yunnan, China | *Liu 500* (SYS) | MG644482 | - |
| *M. fengii* (S.Y. Hu) C.Y. Wu & C. Chen | Pingdong, Taiwan, China | *Liu 534* (SYS) | MG644483 | - |
| *M. longipedunculata* Cogn. | Kuching, Malaysia | *Zhou et al. M35* (SYS) | MG644484 | MG644593 |
| *M. speciosa Blume* | Sabah, Malaysia | *Zhou et al. M2* (SYS) | MG644485 | MG644594 |
| Tr. Sonerileae |  |  |  |  |
| *Allomorphia balansae* Cogn. | Wuzhishan, Hainan, China | *Liu 451* (SYS) | MG644470 | MG644581 |
| *A. baviensis* Guillaumin | Fugong, Yunnan, China | *Q. Fan 13993* (SYS) | MG644471 | MG644582 |
| *A. malaccensis* Ridl. | Kuala Lumpur, Malaysia | *Zhou et al. M44* (SYS) | MG644472 | MG644583 |
| *A. urophylla* Diels | Napo, Guangxi, China | *Liu 488* (SYS) | MG644473 | MG644584 |
| *Anerincleistus macrophyllus* Bakh.f. | Kuching, Malaysia | *Zhou et al. M32* (SYS) | MG644450 | MG644561 |
| *A. phyllagathoides* (Stapf) J.F. Maxwell | Sarawak, Malaysia | *C.W. Lin* 635 (TAIF) | MG993337 | MG993347 |
| *A. quintuplinervis* (Cogn.) J.F. Maxwell | Miri, Malaysia | *Zhou et al. M22* (SYS) | MG644453 | MG644564 |
| *A. sertuliferus* (Cogn.) J.F. Maxwell | Kuching, Malaysia | *Zhou et al. M33* (SYS) | MG644451 | MG644562 |
| *A. setulosus* O. Schwartz | Sabah, Malaysia | *Zhou et al. M11* (SYS) | MG644452 | MG644563 |
| *Barthea barthei* (Hance ex Benth.) Krasser | Yangchun, Guangdong, China | *Liu 575* (SYS) | MG644388 | MG644499 |
| *Blastus apricus* (Hand.-Mazz.) H.L. Li | Huaiji, Guangdong, China | *Liu 542* (SYS) | MG644463 | MG644574 |
| *B. cavaleriei* H. Lév. & Vaniot | Dongan, Hunan, China | *Liu 461* (SYS) | MG644464 | MG644575 |
| *B. cochinchinensis* Lour. | Fengkai, Guandong, China | *Liu 446* (SYS) | MG644465 | MG644576 |
| *B. cochinchinensis* Lour. | Yilan, Taiwan | *Liu 528* (SYS) | MG644466  MG644467 | MG644577  MG644578 |
| *B. ernae* Hand.-Mazz. | Rucheng, Hunan, China | *Liu 469* (SYS) | MG644468 | MG644579 |
| *B. mollissimus* H.L. Li | Guiping, Guangxi, China | *Liu 555* (SYS) | MG644469 | MG644580 |
| *Bredia amoena* Diels | Zixi, Jiangxi, China | *Liu 545* (SYS) | MG644389 | MG644500 |
| *B. amoena* Diels | Pingnan, Fujian, China | *Liu 571* (SYS) | MG644390 | MG644501 |
| *B. biglandularis* C. Chen | Fangcheng, Guangxi, China | *Liu 553* (SYS) | MG644392 | MG644503 |
| *B. changii* W.Y. Zhao, X.H. Zhan & W.B. Liao | Chongyi, Jiangxi, China | *Liu 548* (SYS) | MF952714 | MF952717 |
| *B. dulanica* C.L. Yeh, S.W. Chung & T.C. Hsu | Dulanshan, Taidong, Taiwan | *Liu 543* (SYS) | MG644414 | MG644525 |
| *B. esquirolii* (H. Lév.) Lauener | Chishui, Guizhou, China | *Liu 587* (SYS) | MG644399 | MG644510 |
| *B. gibba* Ohwi | Pingdong, Taiwan | *Liu 566* (SYS) | MG644415 | MG644526 |
| *B. hirsuta* Blume | Komi, Iriomote, Japan | *Alison Wee & Akiyo Naiki 8032* (SYS) | MG644416 | MG644527 |
| *B. hirsuta* Blume | Taidong, Taiwan | *Liu 563* (SYS) | MG644417 | MG644528 |
| *B. hirsuta* Blume var. *scandens* Ito & Matsum. | Nantou, Taiwan | *Liu 539* (SYS) | MG644420 | MG644531 |
| *B. longiloba* (Hand.-Mazz.) Diels | Zixi, Jiangxi, China | *Liu 544* (SYS) | MF952715 | MF952718 |
| *B. microphylla* H.L. Li | Longsheng, Guangxi, China | *Liu 551* (SYS) | MF952716 | MF952719 |
| *B. okinawensis* (Matsumura) H.L.Li (= *Tashiroea okinawensis* Matsum.) | Okinawa, Japan | *Liu 636* (SYS) | MK951697* | MK953743* |
| *B. oldhamii* Hook. f. | Taidong, Taiwan | *Liu 564* (SYS) | MG644418 | MG644529 |
| *B. quadrangularis* Cogn. | Guidong, Hunan, China | *Liu 473* (SYS) | MG644391 | MG644502 |
| *B. repens* R.C. Zhou, Q.J. Zhou & Y. Liu | Sangzhi, Hunan, China | *Liu 558* (SYS) | MF952713 | MF952720 |
| *B. rotundifolia* Y.C. Liu & C.H. Ou | Zhanghua, Taiwan | *Liu 538* (SYS) | MG644419 | MG644530 |
| *B. sessilifolia* H.L. Li | Huaiji, Guangdong, China | *Liu 540* (SYS) | MG644393 | MG644504 |
| *B. sinensis* (Diels) H.L. Li (= *Tashiroea sinensis* Diels) | Zixi, Jiangxi, China | *Liu 546* (SYS) | MG644397 | MG644508 |
| *B. sinensis* (Diels) H.L. Li (= *Tashiroea sinensis* Diels) | Pingnan, Fujian, China | *Liu 569* (SYS) | MG644398 | MG644509 |
| *B. tuberculata* (Guillaumin) Diels | Emei, Sichuan, China | *Liu 579* (SYS) | MG644412 | MG644523 |
| *B. yaeyamensis* (Matsum.) H.L. Li (= *Tashiroea yaeyamensis* Matsum.) | Komi, Iriomote, Japan | *Alison Wee & Akiyo Naiki 8024* (SYS) | MG644396 | MG644507 |
| *B. yunnanensis* (H.Lév.) Diels | Shuigu, Yunnan, China | *Liu 627 (SYS)* | MK951702* | MK953746* |
| *Cyphotheca montana* Diels | Jinping, Yunnan, China | *Liu 596* (SYS) | MG644447 | MG644558 |
| *Driessenia axantha* Korth. | Kuching, Malaysia | *Zhou et al. M26* (SYS) | MG644454 | MG644565 |
| *D. glanduligera* Stapf | Sabah, Malaysia | *Zhou et al. 657* (SYS) | MK951698* | MK953740* |
| *D. phasmolacuna* C.W. Lin | Sarawak, Malaysia | *C.W. Lin 659* (SYS) | MK951699* | MK953745* |
| *Fordiophyton breviscapum* (C. Chen) Y.F. Deng & T.L. Wu | Ruyuan, Guangdong, China | *Liu 441* (SYS) | MG644455 | MG644566 |
| *F. faberi* Stapf | Guidong, Hunan, China | *Liu 474* (SYS) | MG644457 | MG644568 |
| *F. faberi* Stapf | Linchuan, Guangxi, China | *Liu 552* (SYS) | MG644456 | MG644567 |
| *F. huizhouensis* S.J. Zeng & X.Y. Zhuang | Huidong, Guangdong, China | *Liu 433* (SYS) | MG644458 | MG644569 |
| *F. peperomiifolium* (Oliv.) C. Hansen | Qingyuan, Guangdong, China | *Liu 432* (SYS) | MG644459 | MG644570 |
| *F. repens* Y.C. Huang ex C. Chen | Pingbian, Yunnan, China | *Liu 513* (SYS) | MG644460 | MG644571 |
| *F. strictum* Diels | Tianlin, Guangxi, China | *Liu 494* (SYS) | MG644461 | MG644572 |
| *F. zhuangiae* S.J. Zeng & G.D. Tang | Yangchuan, Guangdong, China | *Liu 574* (SYS) | MG644462 | MG644573 |
| *Oxyspora paniculata* DC. | Napo, Guangxi, China | *Liu 493* (SYS) | MG644475 | MG644586 |
| *Oxyspora teretipetiolata* (C.Y. Wu & C. Chen) W.H. Chen & Y.M. Shui | Jinping, Yunnan, China | *Liu 598* (SYS) | MG644476 | MG644587 |
| *Phyllagathis cavaleriei* (H. Lév. & Vaniot) Guillaumin | Ruyuan, Guangdong, China | *Liu 438* (SYS) | MG644421 | MG644532 |
| *P. cavaleriei* (H. Lév. & Vaniot) Guillaumin | Dongkou, Hunan, China | *Liu 456* (SYS) | MG644422 | MG644533 |
| *P. cavaleriei* (H. Lév. & Vaniot) Guillaumin | Napo, Guangxi, China | *Liu 487* (SYS) | MG644423 | MG644534 |
| *P. cavaleriei* var. *wilsoniana* Guillaumin | Hongya, Sichuan, China | *Liu 599* (SYS) | MG993330 | MG993340 |
| *P. cymigera* C. Chen | Malipo, Yunnan, China | *E.D. Liu et al. 5117* (KUN) | MG993338 | MG993348 |
| *P. dispar* (Cogn.) C. Hansen | Sabah, Malaysia | *Zhou et al. M20* (SYS) | MG644429 | MG644540 |
| *P. elattandra* Diels | Guiping, Guangxi, China | *Liu 554* (SYS) | MG644431 | MG644542 |
| *P. elliptica* Stapf | Sabah, Malaysia | *Zhou et al. M9* (SYS) | MG644430 | MG644541 |
| *P. erecta* (S.Y. Hu) C.Y. Wu ex C. Chen | Malipo, Yunnan, China | *Liu 507* (SYS) | MG644442 | MG644553 |
| *P. fengii* C. Hansen | Xichou, Yunnan, China | *Liu 520* (SYS) | MG644448 | MG644559 |
| *P. fordii* (Hance) C.Chen | Fengkai, Guangdong, China | *Liu 444* (SYS) | MG644400 | MG644511 |
| *P. fordii* (Hance) C.Chen var. *micrantha* C.Chen | Leshan, Sichuan, China | *Liu 580* (SYS) | MG644401 | MG644512 |
| *P. gigantifolia* M.P. Nayar | Sabah, Malaysia | *Zhou et al. M10* (SYS) | MG644433 | MG644544 |
| *P. gracilis* (Hand.-Mazz.) C. Chen | Dongkou, Hunan, China | *Liu 455* (SYS) | MG644402 | MG644513 |
| *P. gracilis* (Hand.-Mazz.) C. Chen | Wugang, Hunan, China | *Liu 457* (SYS) | MG644403 | MG644514 |
| *P. griffithii* King | Selangor, Malaysia | *Liu 600* (SYS) | MG993331 | MG993341 |
| *P. guidongensis* K.M. Liu & J. Tian | Guidong, Hunan, China | *Liu 472* (SYS) | MG644404 | MG644515 |
| *P. gymnantha* Korth. | Kuching, Malaysia | *Zhou et al. M31* (SYS) | MG644434 | MG644545 |
| *P. gymnantha* Korth. | Sarawak, Malaysia | *C.W. Lin 625* (TAIF) | MG993334 | MG993344 |
| *P. hainanensis* (Merr. & Chun) C. Chen | Lingshui, Hainan, China | *Liu 448* (SYS) | MG644439 | MG644550 |
| *P. hispida* King | Kuala Lumpur, Malaysia | *Zhou et al. M49* (SYS) | MG644435 | MG644546 |
| *P. hispidissima* (C.Chen) C.Chen | Pingbian, Yunnan, China | *Liu 604* (SYS) | MK951695* | MK953744* |
| *P. latisepala* C. Chen | Sangzhi, Hunan, China | *Liu 557* (SYS) | MG644405 | MG644516 |
| *P. longearistata* C. Chen | Huanjiang, Guangxi, China | *Liu 498* (SYS) | MG644406 | MG644517 |
| *P. longiradiosa* (C. Chen) C. Chen | Napo, Guangxi, China | *Liu 492* (SYS) | MG644407  MG644408 | MG644518  MG644519 |
| *P. longiradiosa* var. *pulchella* C. Chen | Longzhou, Guangxi, China | *Liu 485* (SYS) | MG644409 | MG644520 |
| *P. melastomatoides* (Merr. & Chun) W.C. Ko | Lingshui, Hainan, China | *Liu 447* (SYS) | MG644424 | MG644535 |
| *P. millelunata* C.W. Lin, Chien F. Chen & T.Y.A. Yang | Sarawak, Malaysia | *C.W. Lin 582* (TAIF) | MG993335 | MG993345 |
| *P. nudipes* C. Chen | Ruyuan, Guangdong, China | *Liu 435* (SYS) | MG644394 | MG644505 |
| *P. oligotricha* Merr. | Rucheng, Hunan, China | *Liu 468* (SYS) | MG644395 | MG644506 |
| *P. osmantha* (M.P. Nayar) Cellin. | Sarawak, Malaysia | *C.W. Lin 567* (TAIF) | MG993336 | MG993346 |
| *P. ovalifolia* H.L. Li | Pingbian, Yunnan, China | *Liu 512* (SYS) | MG644425 | MG644536 |
| *P. plagiopetala* C. Chen | Xinning, Hunan, China | *Liu 459* (SYS) | MG644410 | MG644521 |
| *P. plagiopetala* C. Chen | Dongan, Hunan, China | *Liu 460* (SYS) | MG644411 | MG644522 |
| *P. postrata* C. Hansen | Gia Lai Province, Vietnam | *C.W. Lin 640* (TAIF) | MG993332 | MG993342 |
| *P. rajah* C.W. Lin, Chien F. Chen & T.Y.A. Yang | Sarawak, Malaysia | *C.W. Lin 644* (TAIF) | MG993339 | MG993349 |
| *P. rotundifolia* (Jack) Blume | Kuala Lumpur, Malasysia | *Zhou et al. M50* (SYS) | MG644436 | MG644547 |
| *P. scortechinii* King | Kuala Lumpur, Malasysia | *Zhou et al. M48* (SYS) | MG644437 | MG644548 |
| *P. setotheca* var. *setotuba* C. Chen | Yangchun, Guangzhou, China | *Liu 576* (SYS) | MG644426 | MG644537 |
| *P. stellata* C.W. Lin & C.H. Lee | Sarawak, Malaysia | *C.W. Lin 643* (TAIF) | MG993333 | MG993343 |
| *P. stenophylla* (Merr. & Chun) H.L. Li | Ledong, Hainan, China | *Liu 453* (SYS) | MG644427 | MG644538 |
| *P. tentaculifera* C.Hansen | Jinping, Yunnan, China | *Liu 722 (SYS)* | MK951696* | MK953741* |
| *P. tetrandra* Diels | Xichou, Yunnan, China | *Liu 519* (SYS) | MG644432 | MG644543 |
| *P. tuberculata* King | Kuala Lumpur, Malasysia | *Zhou et al. M43* (SYS) | MG644438 | MG644549 |
| *P. velutina* (Diels) C. Chen | Malipo, Yunnan, China | *Liu 509* (SYS) | MG644413 | MG644524 |
| *P. xinyiensis* Z.J. Feng | Xinyi, Guangdong, China | *Liu 582* (SYS) | MG644428 | MG644539 |
| *Plagiopetalum esquirolii* (H. Lév.) Rehder | Tianlin, Guangxi, China | *Liu 495* (SYS) | MG644445 | MG644556 |
| *P. esquirolii* (H. Lév.) Rehder | Malipo, Yunnan, China | *Liu 504* (SYS) | MG644443  MG644444 | MG644554  MG644555 |
| *Sarcopyramis bodinieri* H.Lév. | Taidong, Taiwan | *Liu 530* (SYS) | MG644486 | MG644595 |
| *S. nepalensis* Wall. | Ruyuan, Guangdong, China | *Liu 437* (SYS) | MG644487 | MG644596 |
| *S. nepalensis* Wall. | Pingdong, Taiwan | *Liu 567* (SYS) | MG644488  MG644489 | MG644597  MG644598 |
| *Scorpiothyrsus oligotrichus* H.L. Li | Ledong, Hainan, China | *Liu 454* (SYS) | MG644440  MG644441 | MG644551  MG644552 |
| *S. shangszeensis* C. Chen | Napo, Guangxi, China | *Liu 626* (SYS) | MK951700* | MK953739* |
| *Sonerila brachyantha* Stapf & King | Kuala Lumpur, Malaysia | *Zhou et al. M40* (SYS) | MG644490 | MG644599 |
| *S. cantonensis* Stapf | Yangchun, Guangdong, China | *Liu 577* (SYS) | MG644492 | MG644601 |
| *S. cantonensis* Stapf | Malipo, Yunnan, China | *Liu 501* (SYS) | MG644493 | MG644602 |
| *S. cantonensis* Stapf | Lingshui, Hainan, China | *Liu 449* (SYS) | MG644491 | MG644600 |
| *S. hainanensis* Merr. | Wuzhishan, Hainan, China | *Liu 450* (SYS) | MG644494 | MG644603 |
| *S. kinabaluensis* Stapf | Sabah, Malaysia | *Zhou et al. M7* (SYS) | MG644495 | MG644604 |
| *S. nervulosa* Ridl. | Sabah, Malaysia | *Zhou et al. M4* (SYS) | MG644496 | MG644605 |
| *S. nidularis* Stapf & King | Kuala Lumpur, Malaysia | *Zhou et al. M47* (SYS) | MG644497 | MG644606 |
| *S. plagiocardia* Diels | Yingde, Guangdong, China | *Liu 443* (SYS) | MG644498 | MG644607 |
| *Sporoxeia latifolia* var. *fengii* (S.Y. Hu) C. Chen | Malipo, Yunnan, China | *Liu 508* (SYS) | MG644446 | MG644557 |
| *S. petelotii* (Merr.) C.Hansen | Jinping, Yunnan, China | *Liu 719* (SYS) | MK951701* | MK953742* |
| *Styrophyton caudatum* (Diels) S.Y. Hu | Napo, Guangxi, China | *Liu 490* (SYS) | MG644449 | MG644560 |
| *Tashiroea villosa* X. X. Su | Pingnan, Fujian, China | *Liu 568* (SYS) | MK967953* | MK978158* |
| *Tigridiopalma magnifica* C. Chen | Gaozhou, Guangdong, China | *Liu 429* (SYS) | MG644449 | MG644560 |
